# Supplementary figures and images for: SplintR ligation-triggered in-situ rolling circle amplification on magnetic bead for accurate detection of circulating microRNAs
Source: PeerJ. 2025 Mar 11;13:e19082. doi: 10.7717/peerj.19082 (PMC11908441; doi:10.7717/peerj.19082)

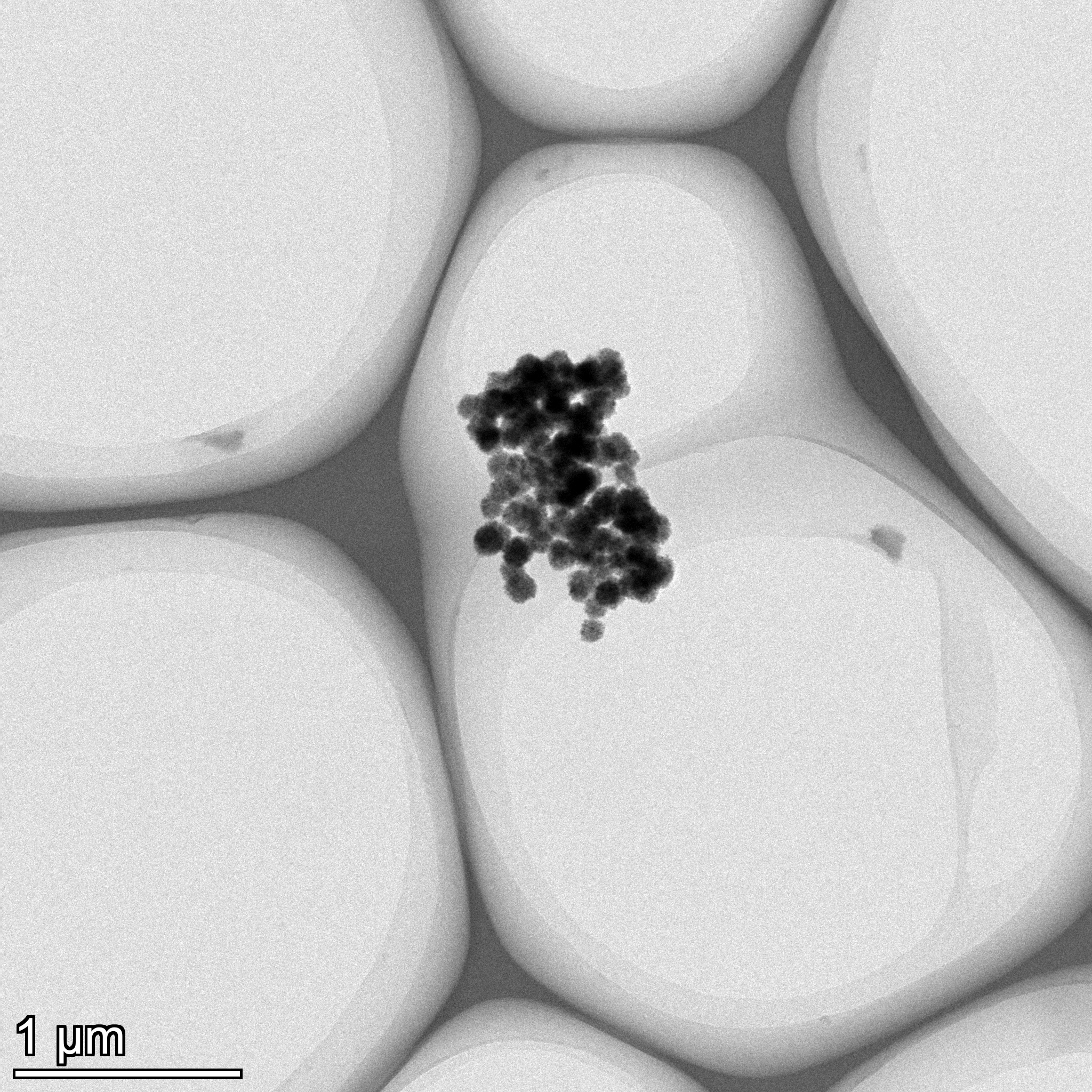

Supplement: Supplemental Information 1 [file peerj-13-19082-s001.jpg]

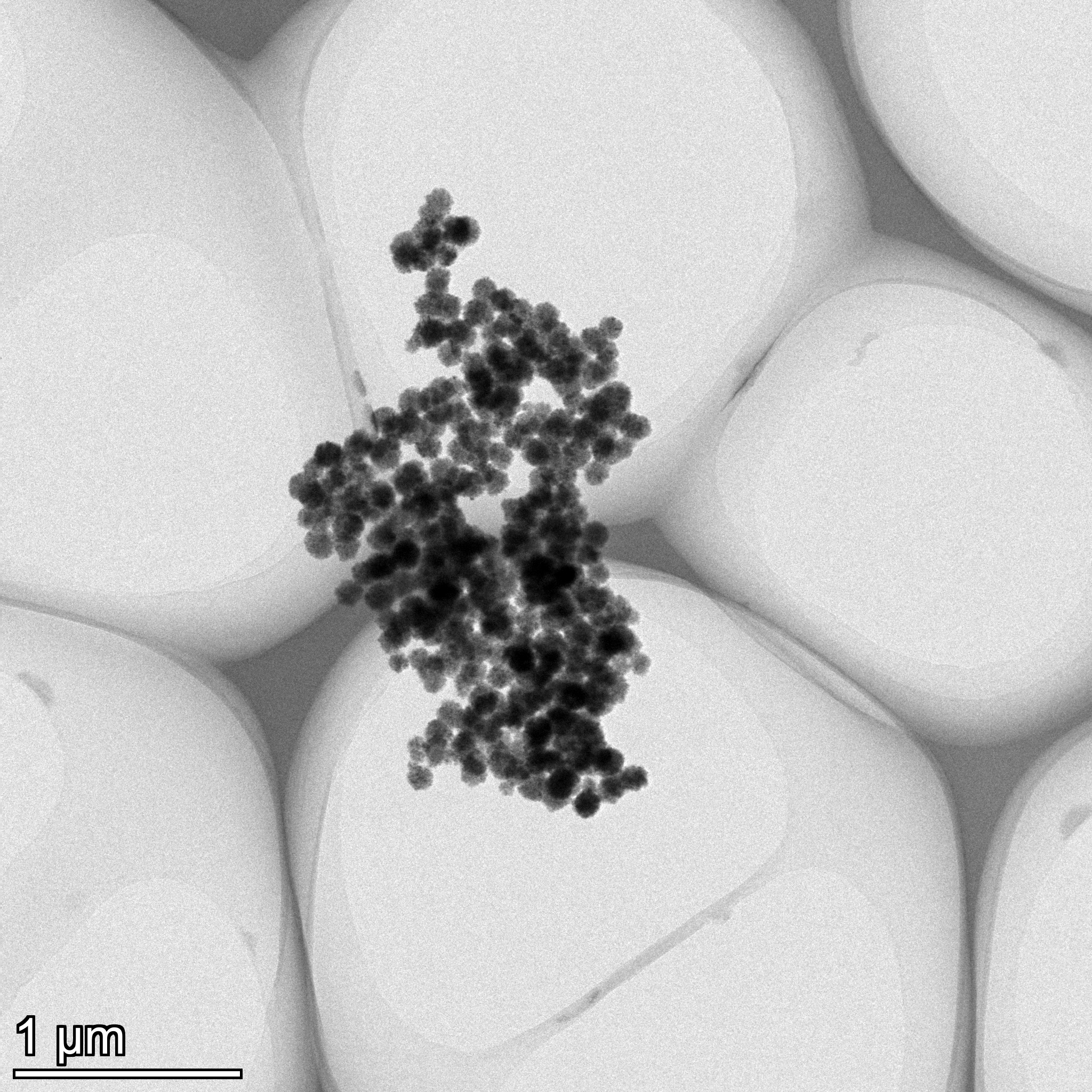

Supplement: Supplemental Information 2 [file peerj-13-19082-s002.jpg]

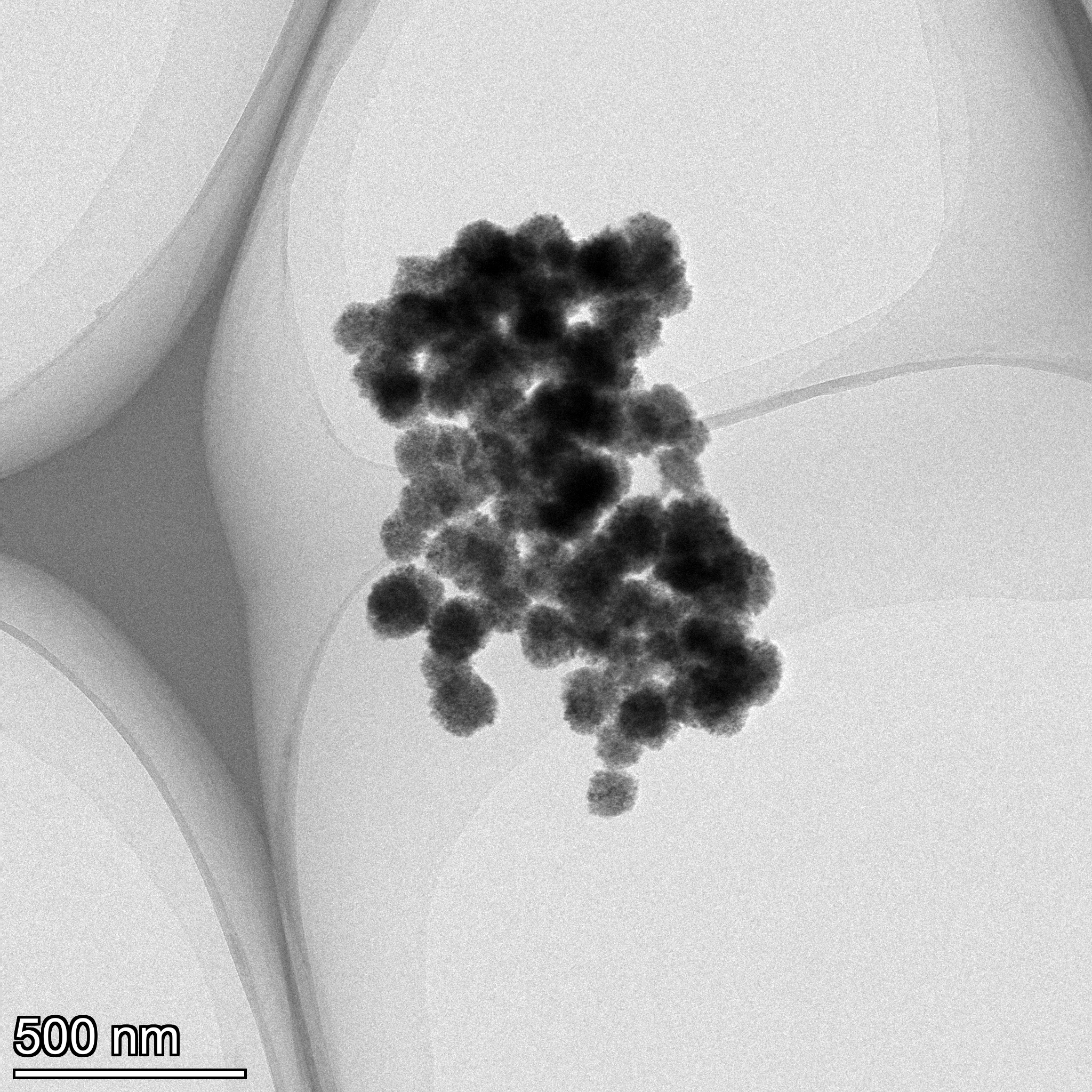

Supplement: Supplemental Information 3 [file peerj-13-19082-s003.jpg]

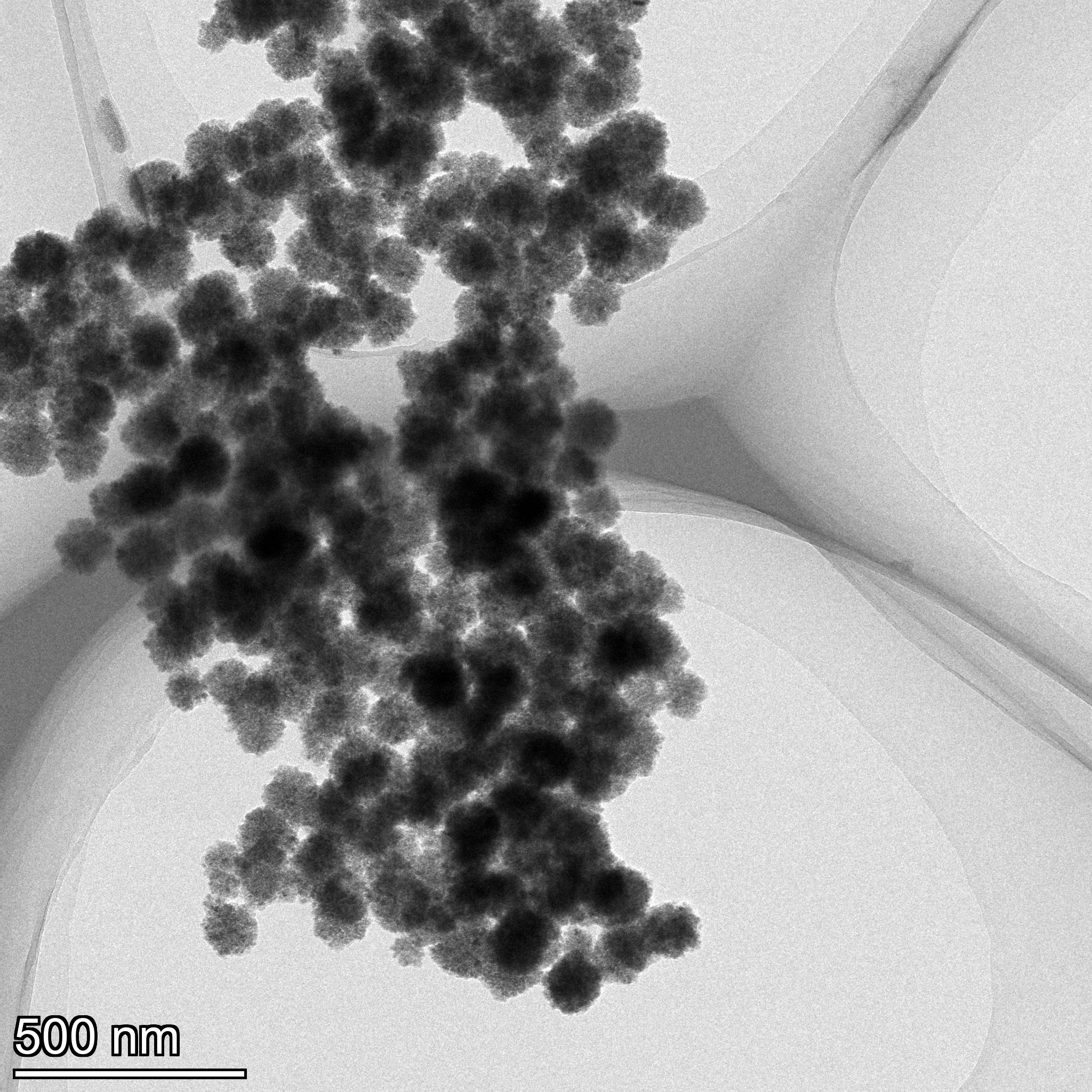

Supplement: Supplemental Information 4 [file peerj-13-19082-s004.jpg]

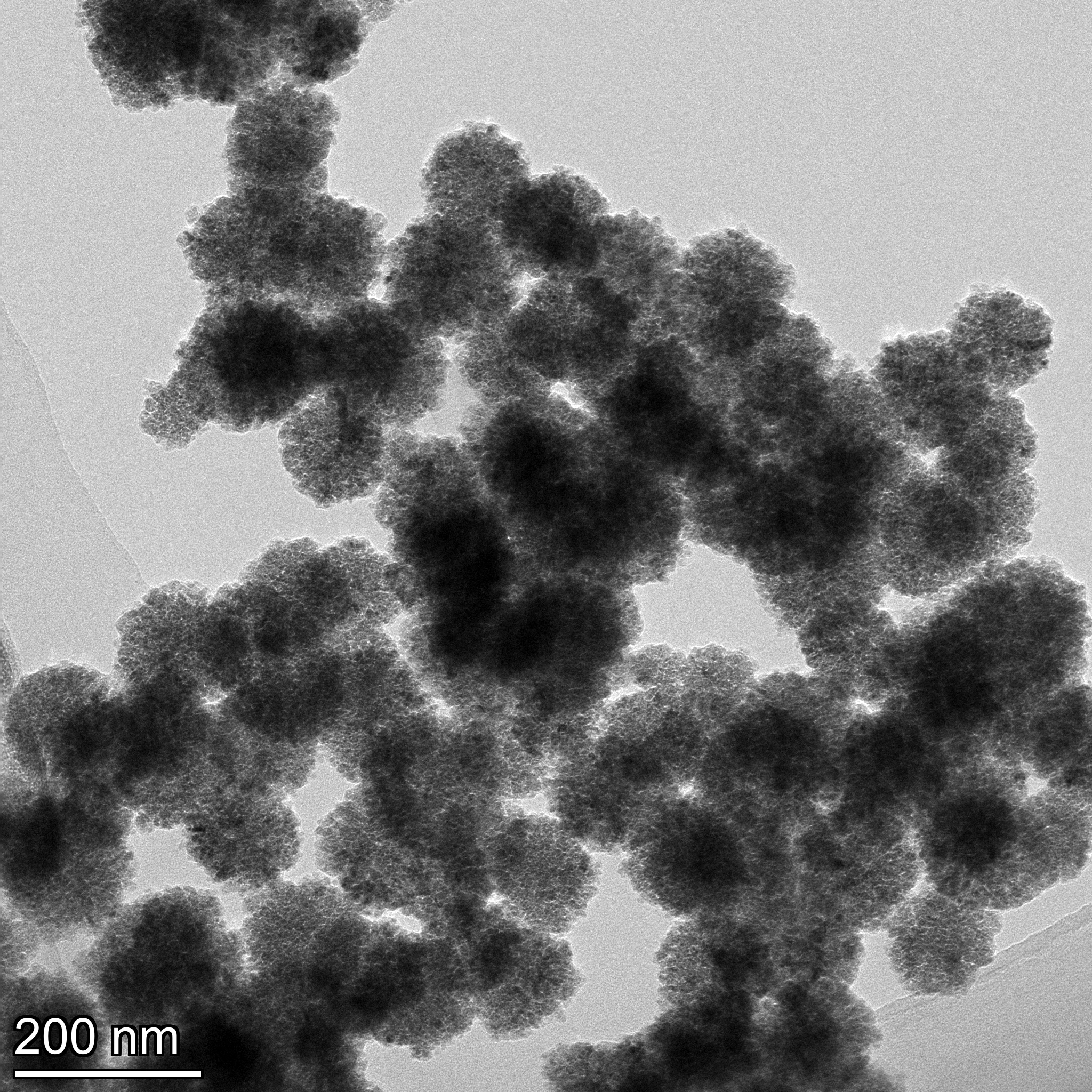

Supplement: Supplemental Information 5 [file peerj-13-19082-s005.jpg]

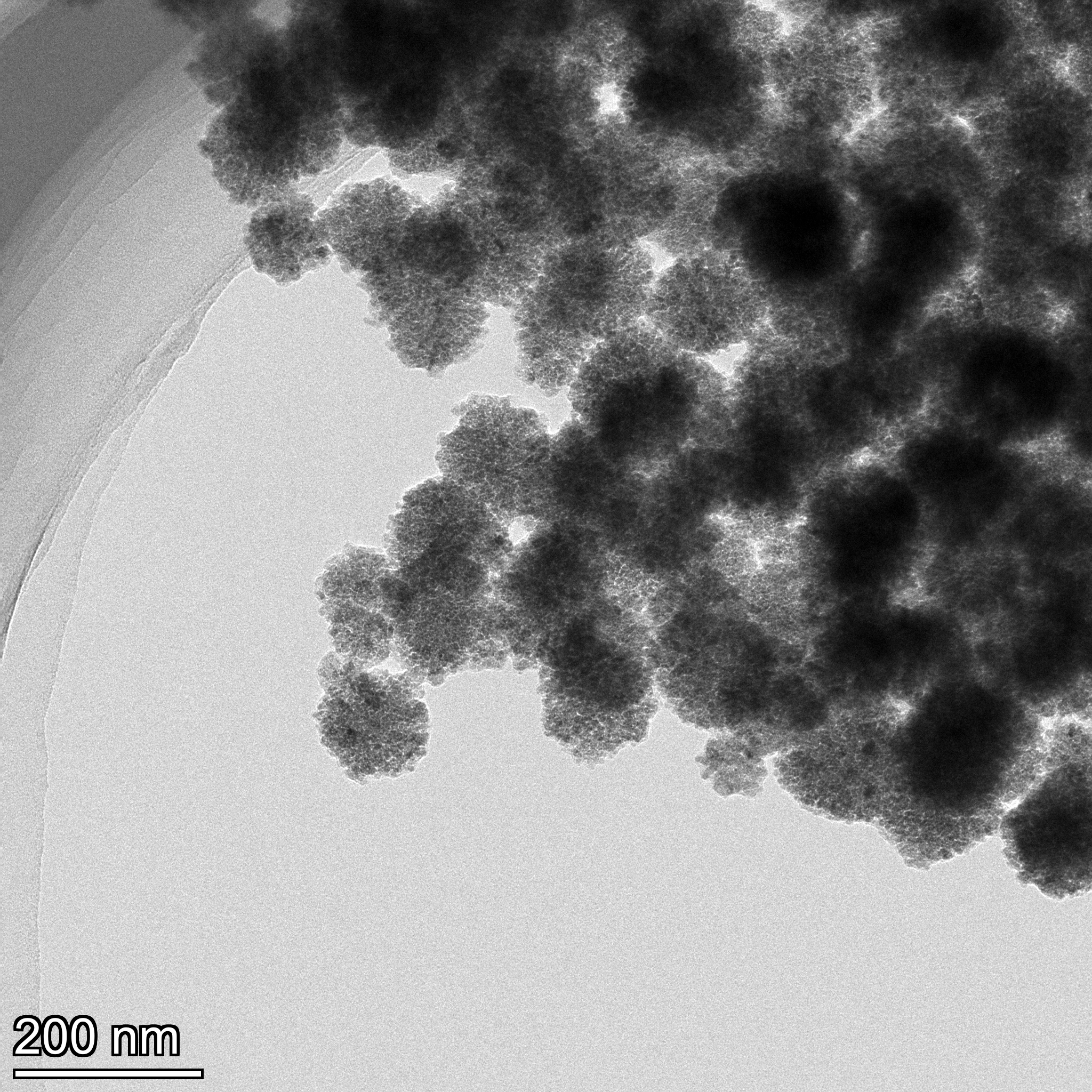

Supplement: Supplemental Information 6 [file peerj-13-19082-s006.jpg]

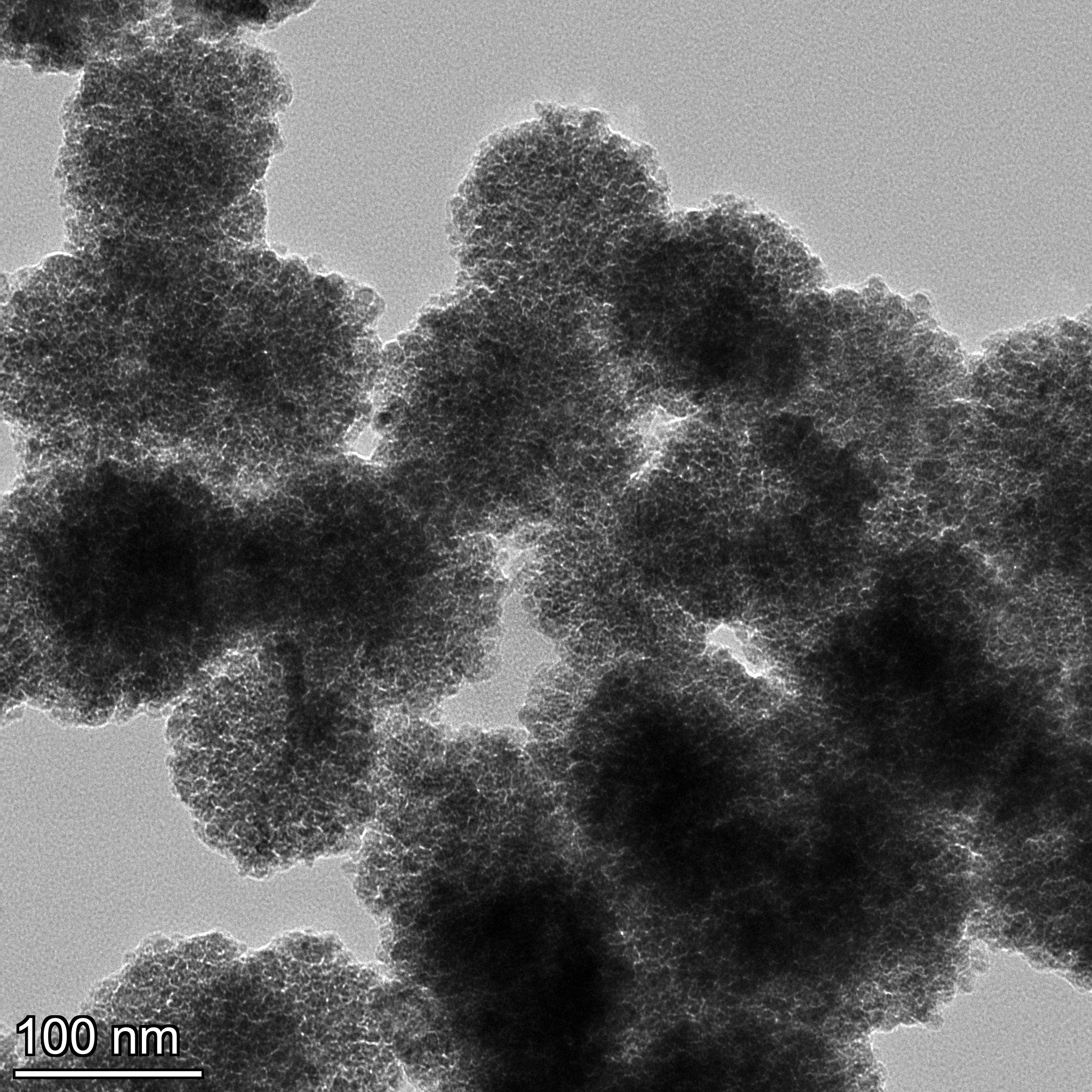

Supplement: Supplemental Information 7 [file peerj-13-19082-s007.jpg]

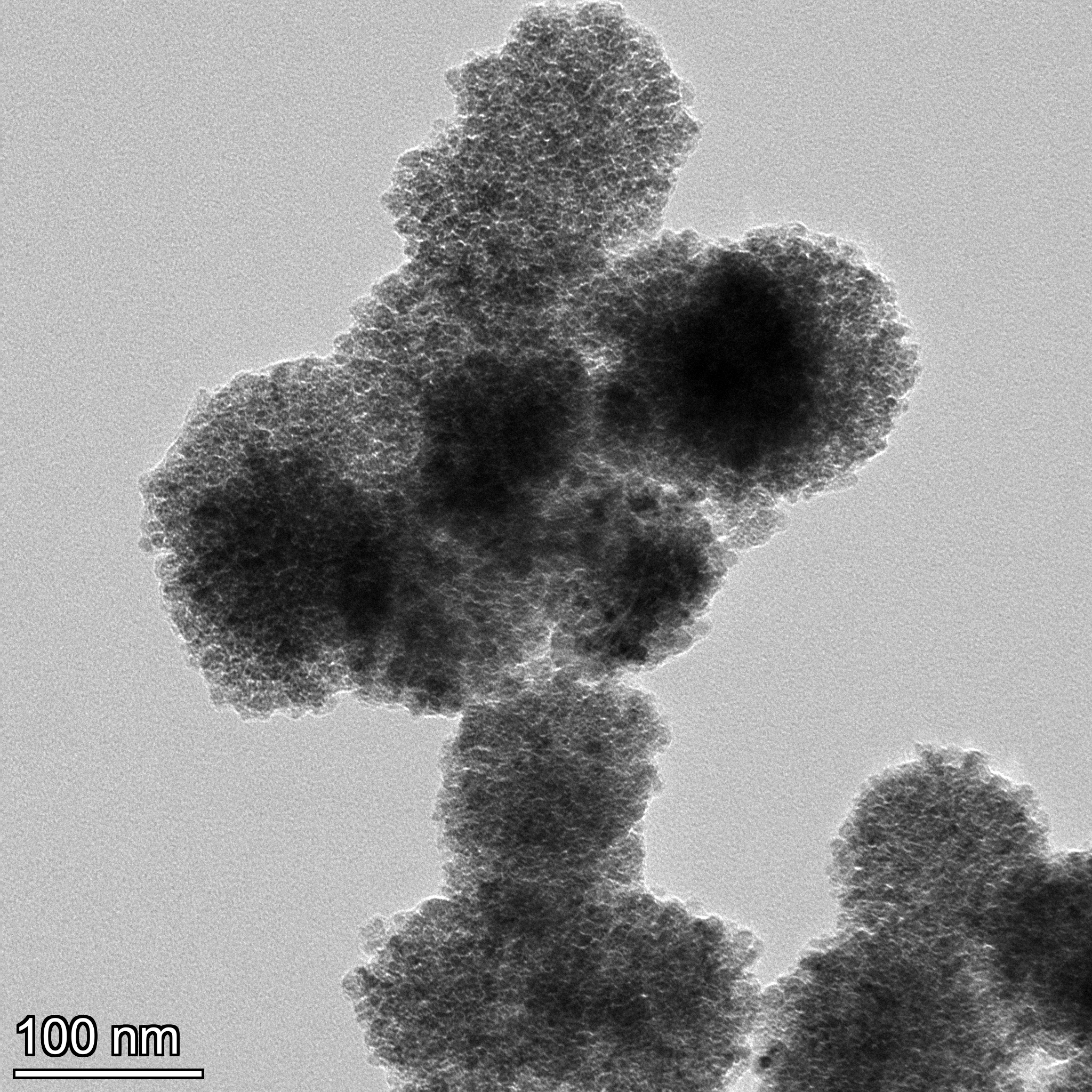

Supplement: Supplemental Information 8 [file peerj-13-19082-s008.jpg]

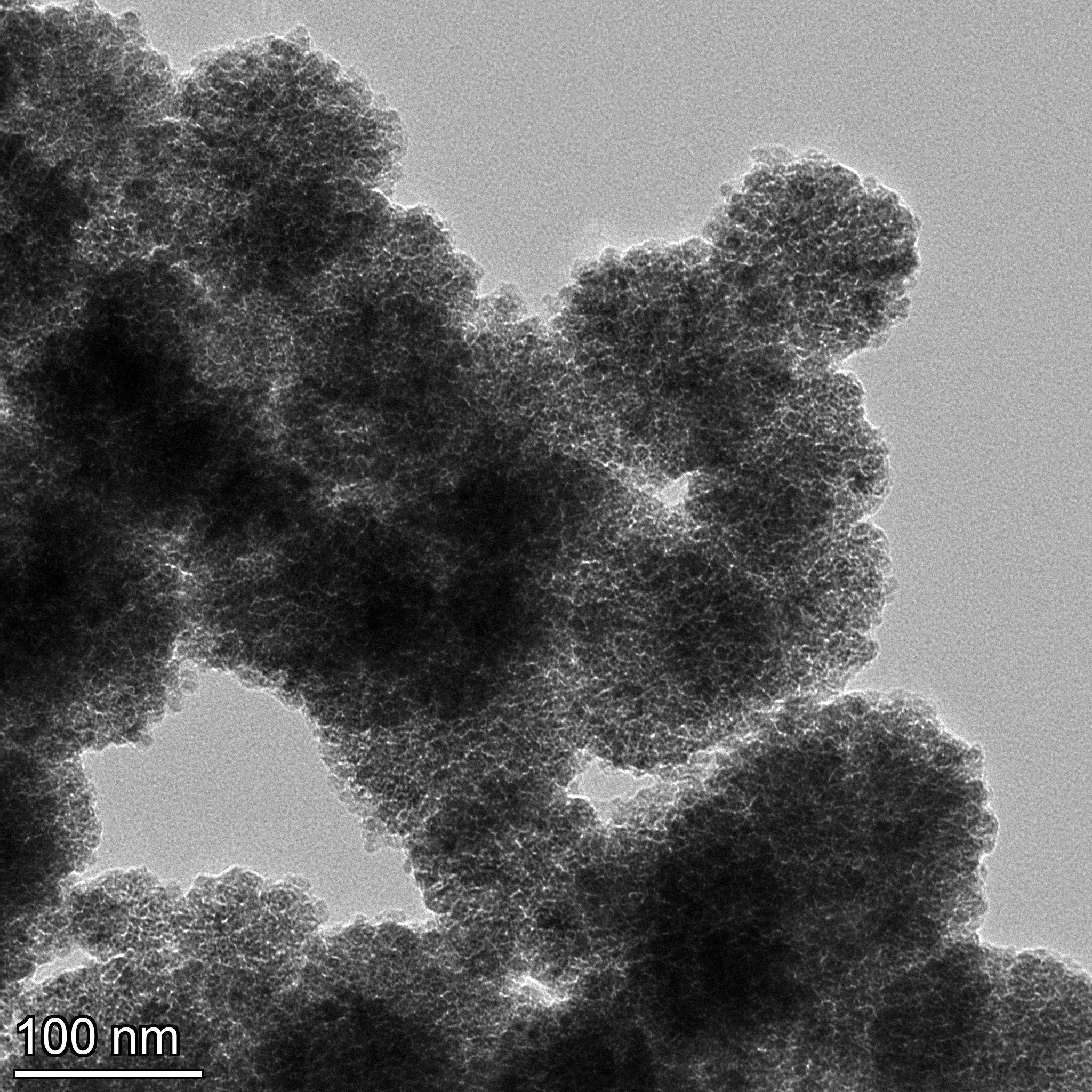

Supplement: Supplemental Information 9 [file peerj-13-19082-s009.jpg]

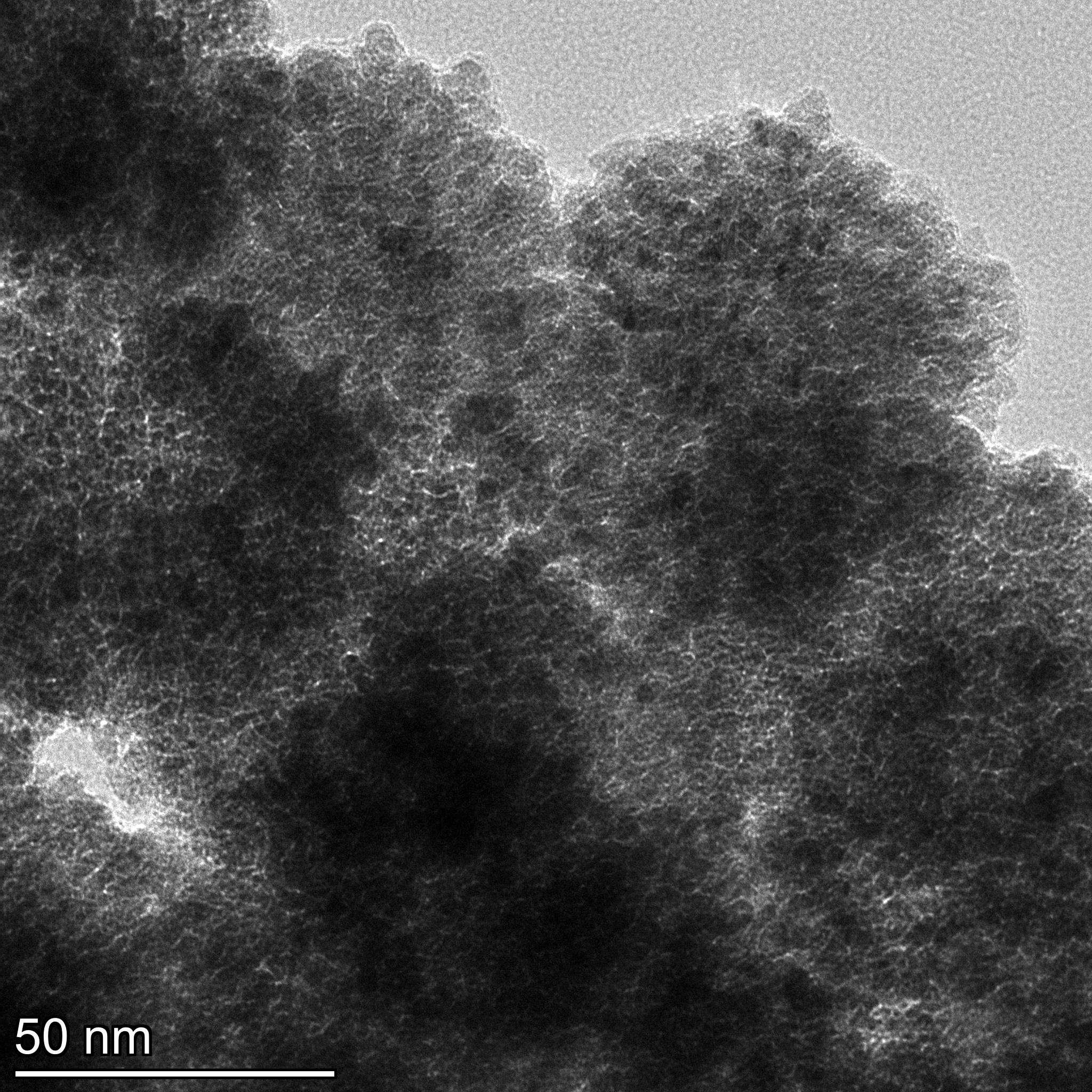

Supplement: Supplemental Information 10 [file peerj-13-19082-s010.jpg]

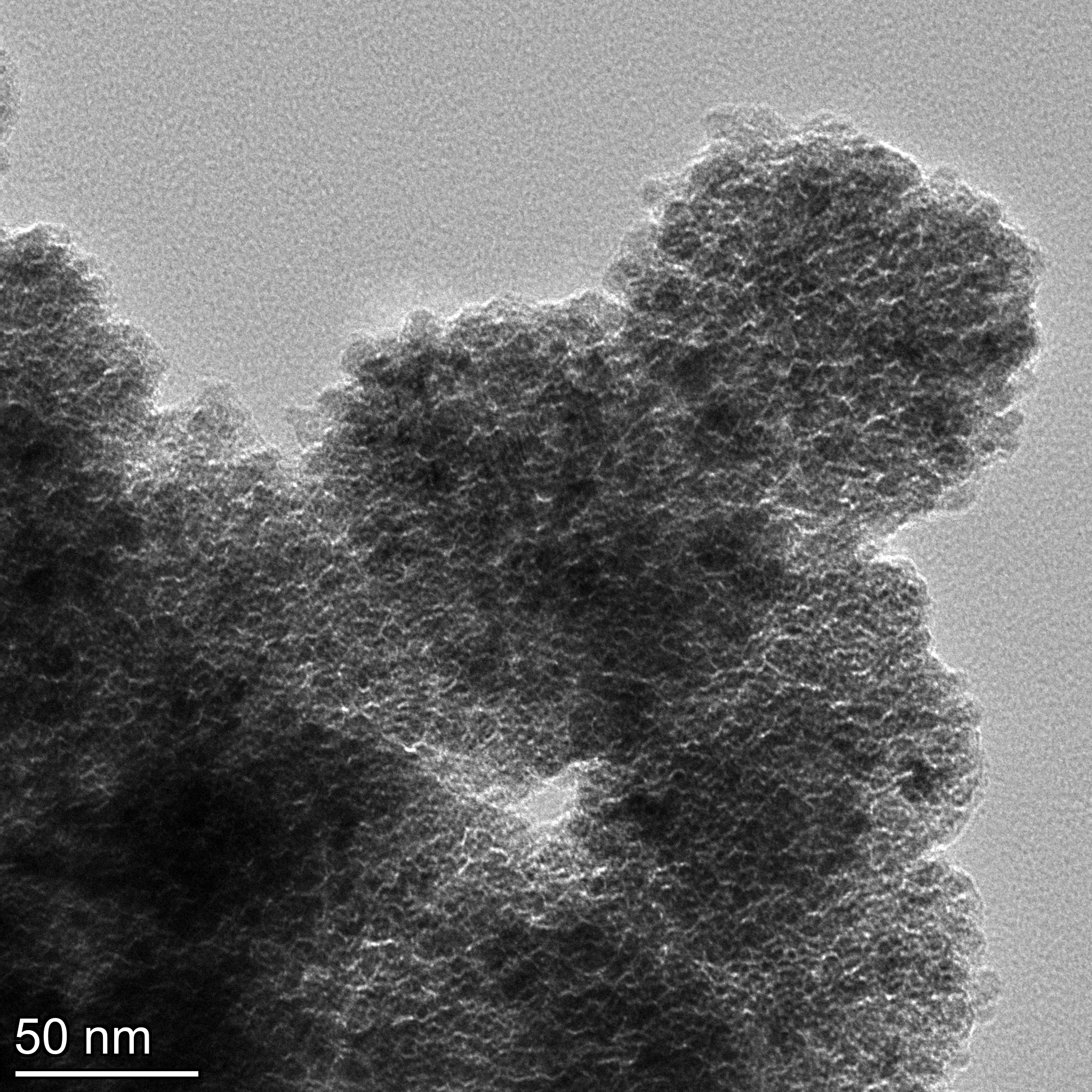

Supplement: Supplemental Information 11 [file peerj-13-19082-s011.jpg]

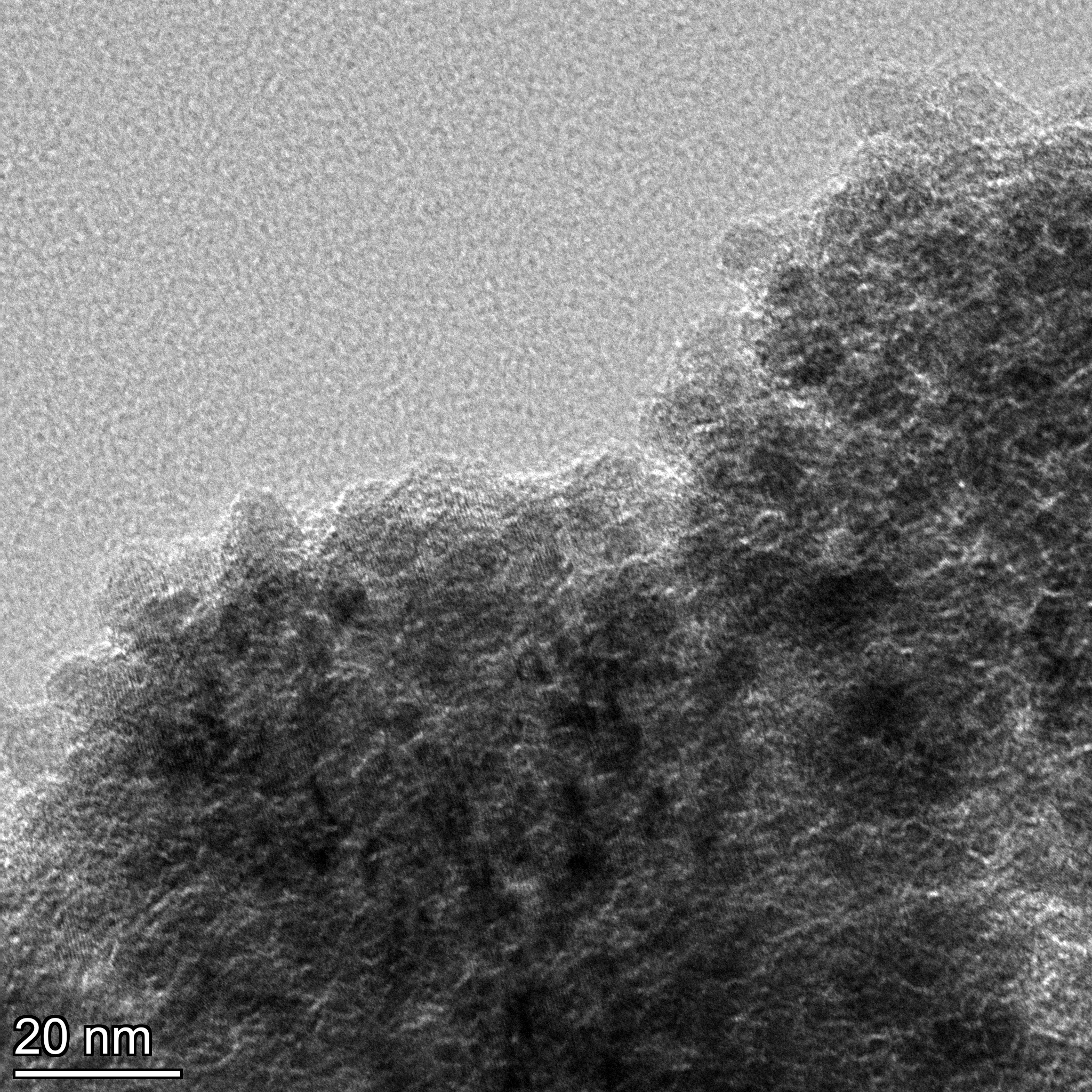

Supplement: Supplemental Information 12 [file peerj-13-19082-s012.jpg]

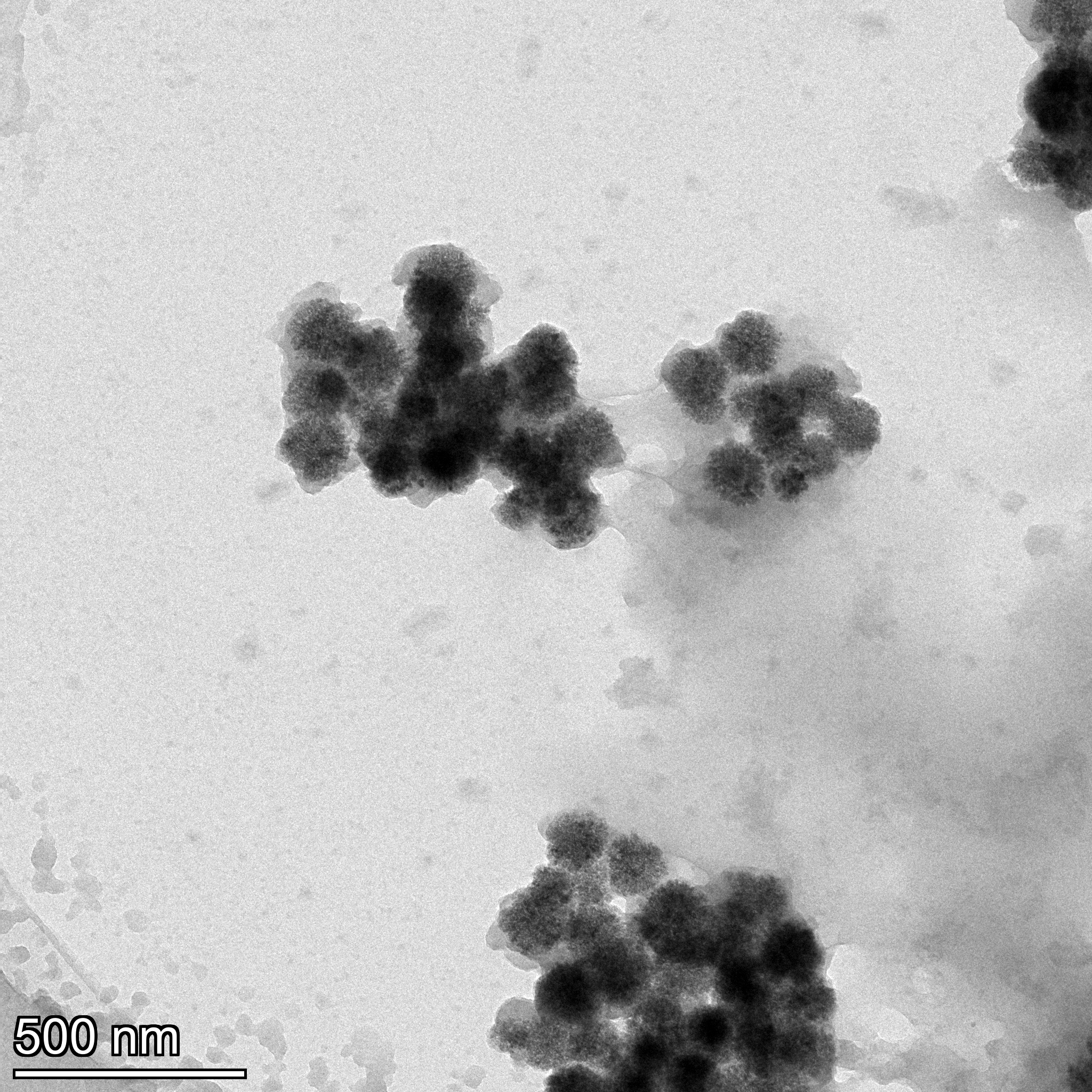

Supplement: Supplemental Information 13 [file peerj-13-19082-s013.jpg]

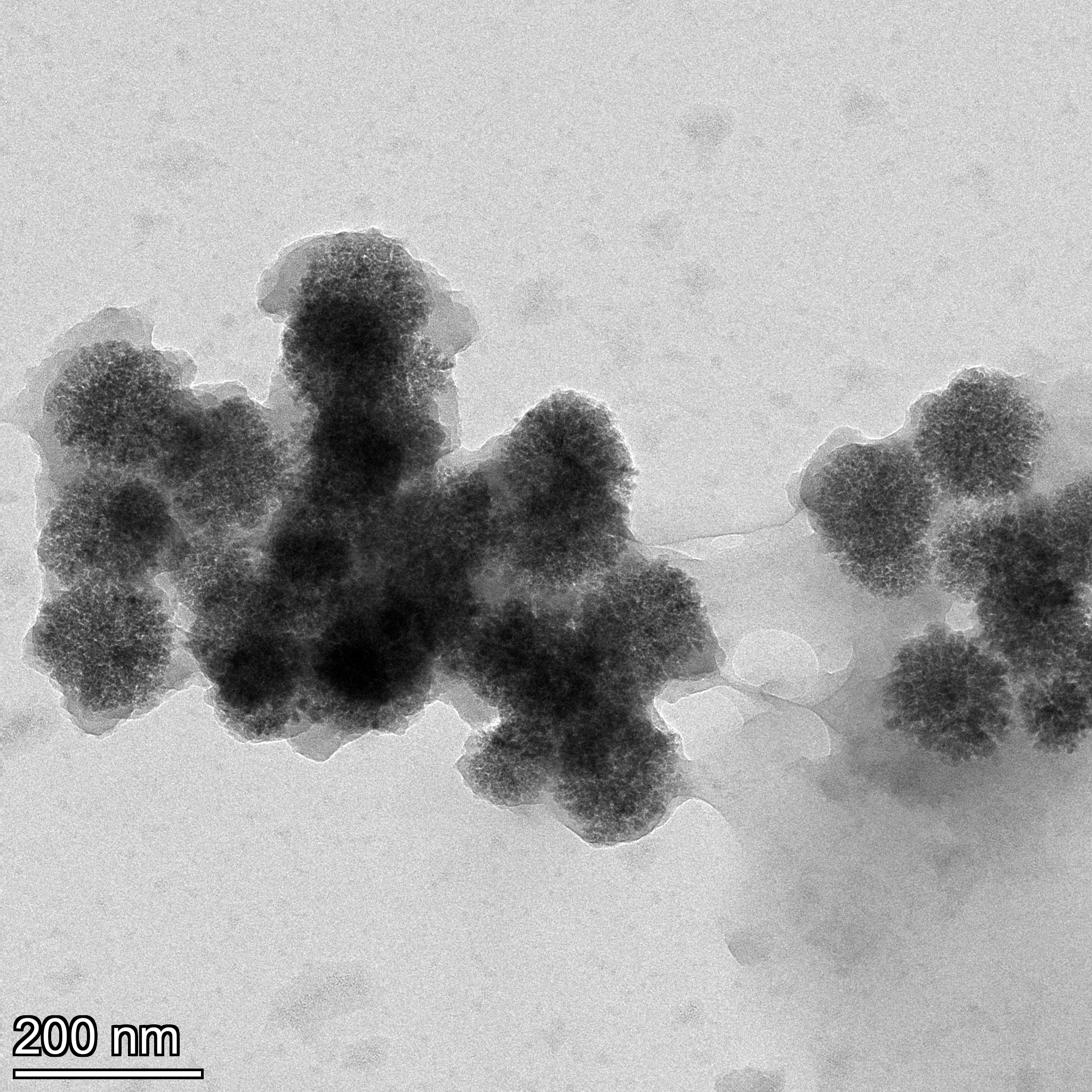

Supplement: Supplemental Information 14 [file peerj-13-19082-s014.jpg]

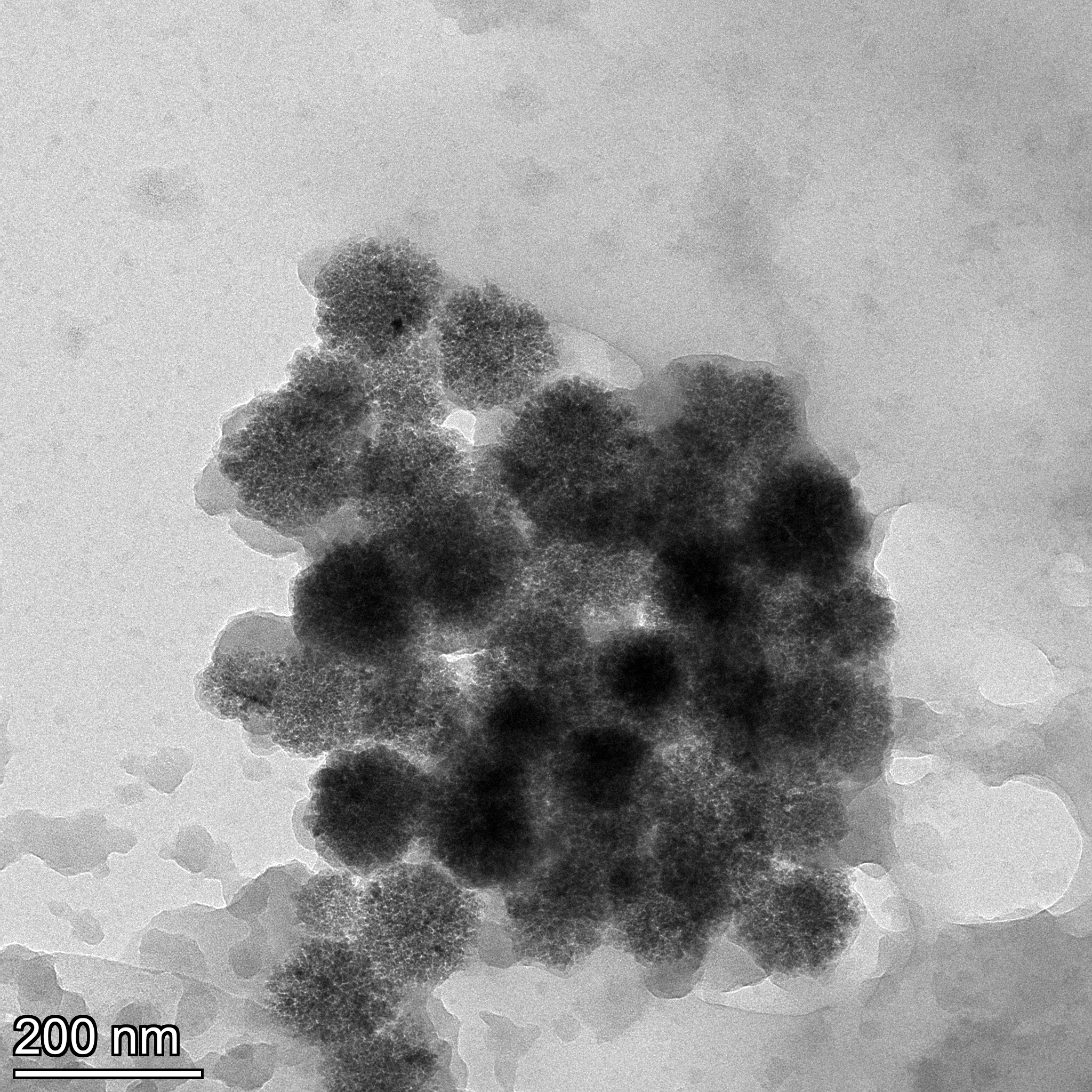

Supplement: Supplemental Information 15 [file peerj-13-19082-s015.jpg]

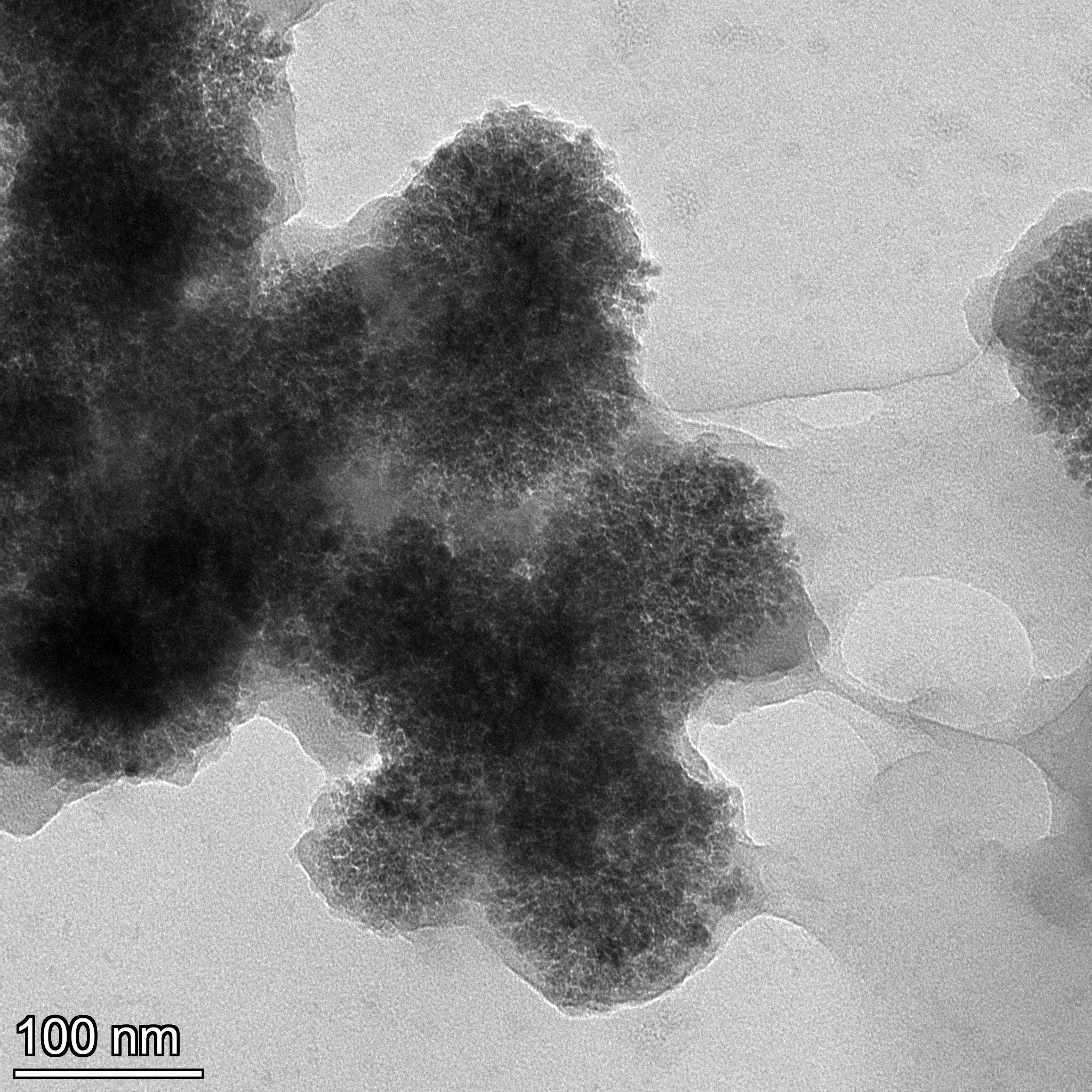

Supplement: Supplemental Information 16 [file peerj-13-19082-s016.jpg]

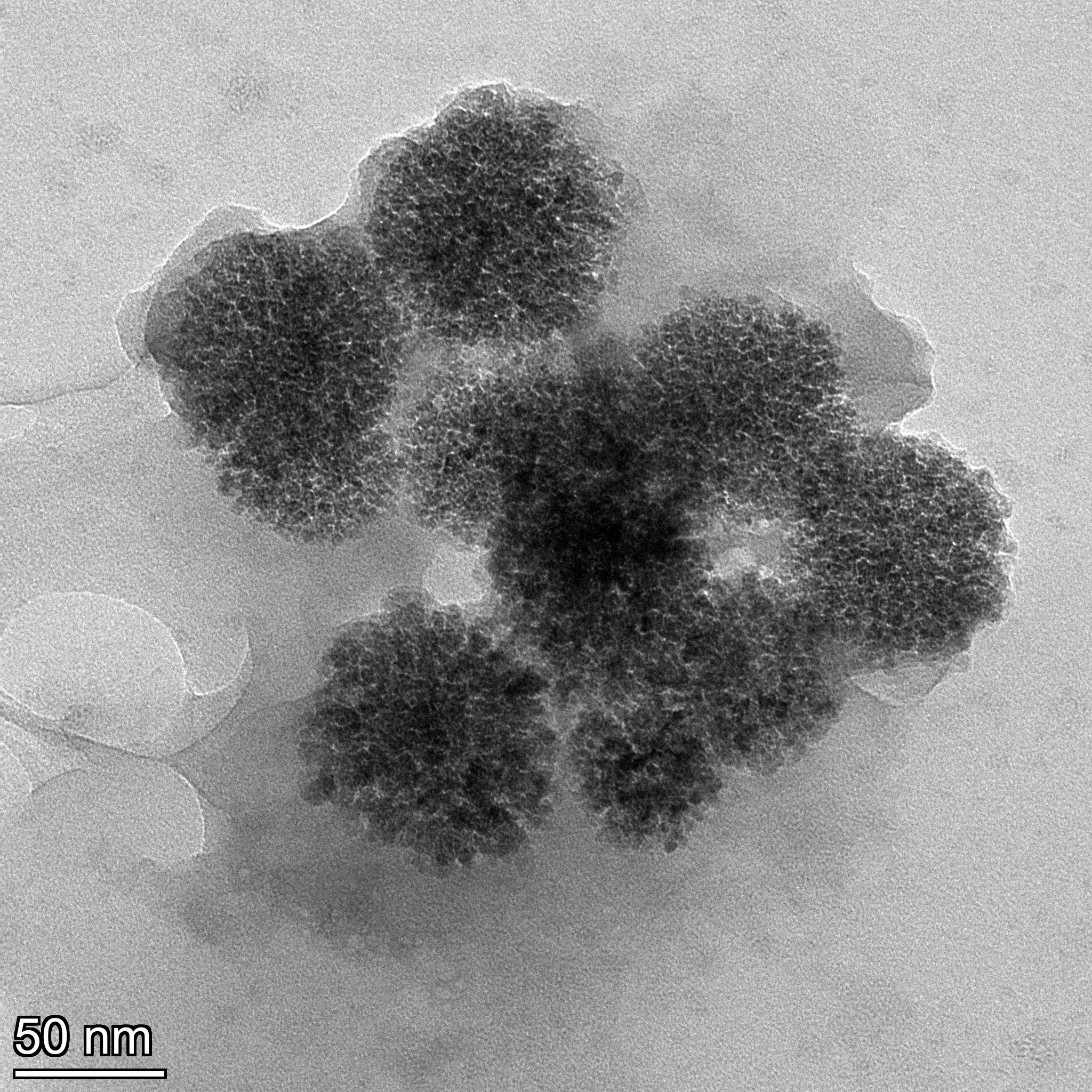

Supplement: Supplemental Information 17 [file peerj-13-19082-s017.jpg]

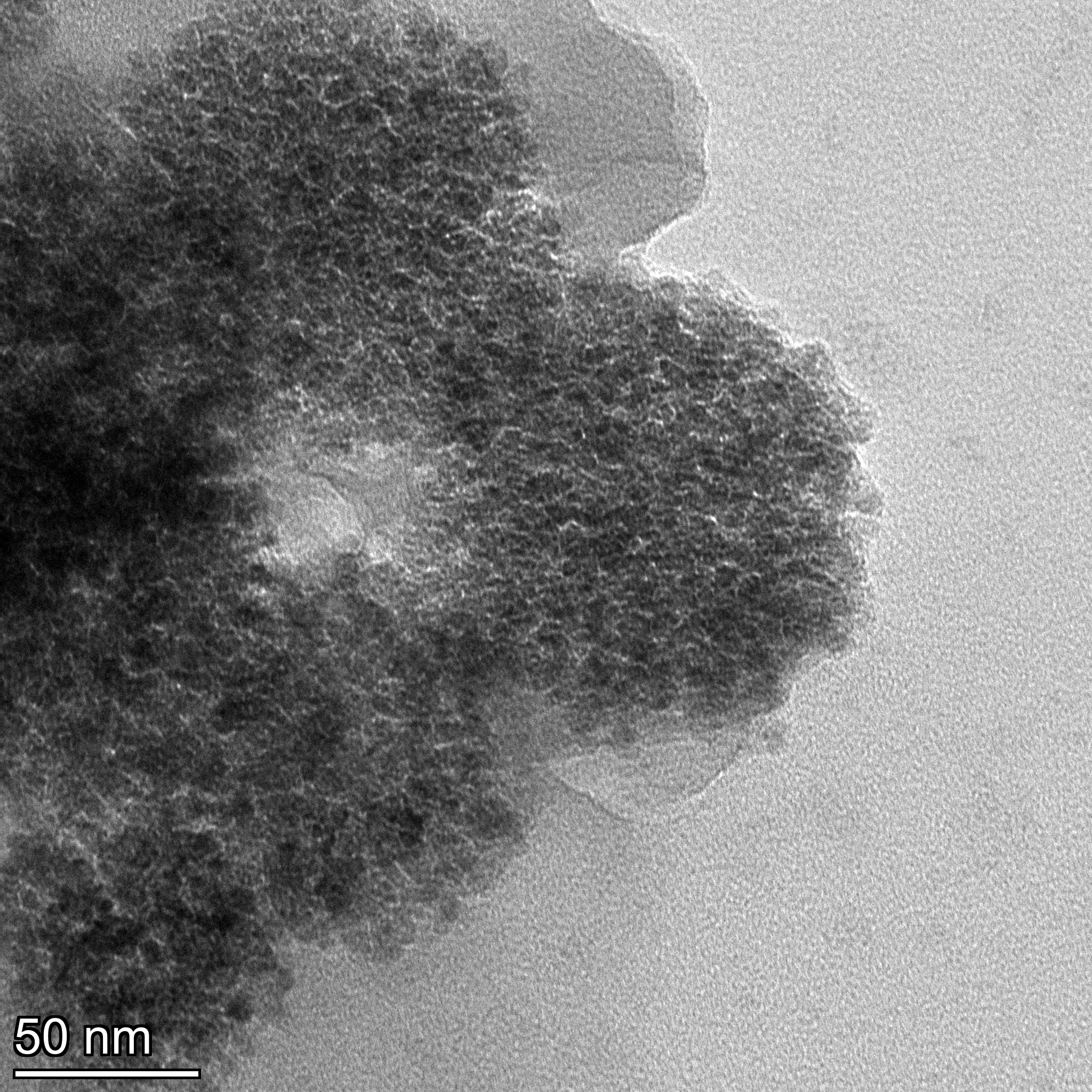

Supplement: Supplemental Information 18 [file peerj-13-19082-s018.jpg]

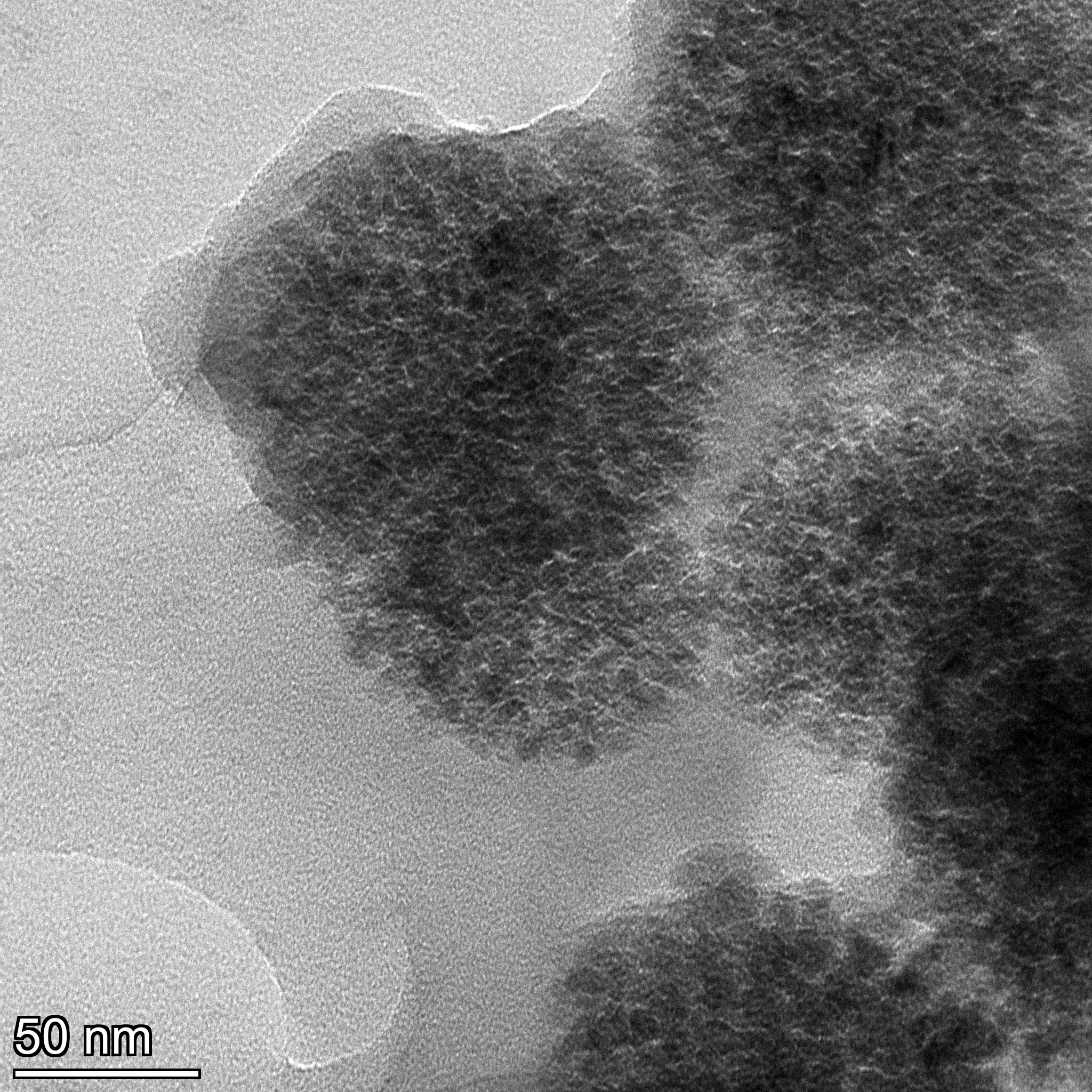

Supplement: Supplemental Information 19 [file peerj-13-19082-s019.jpg]

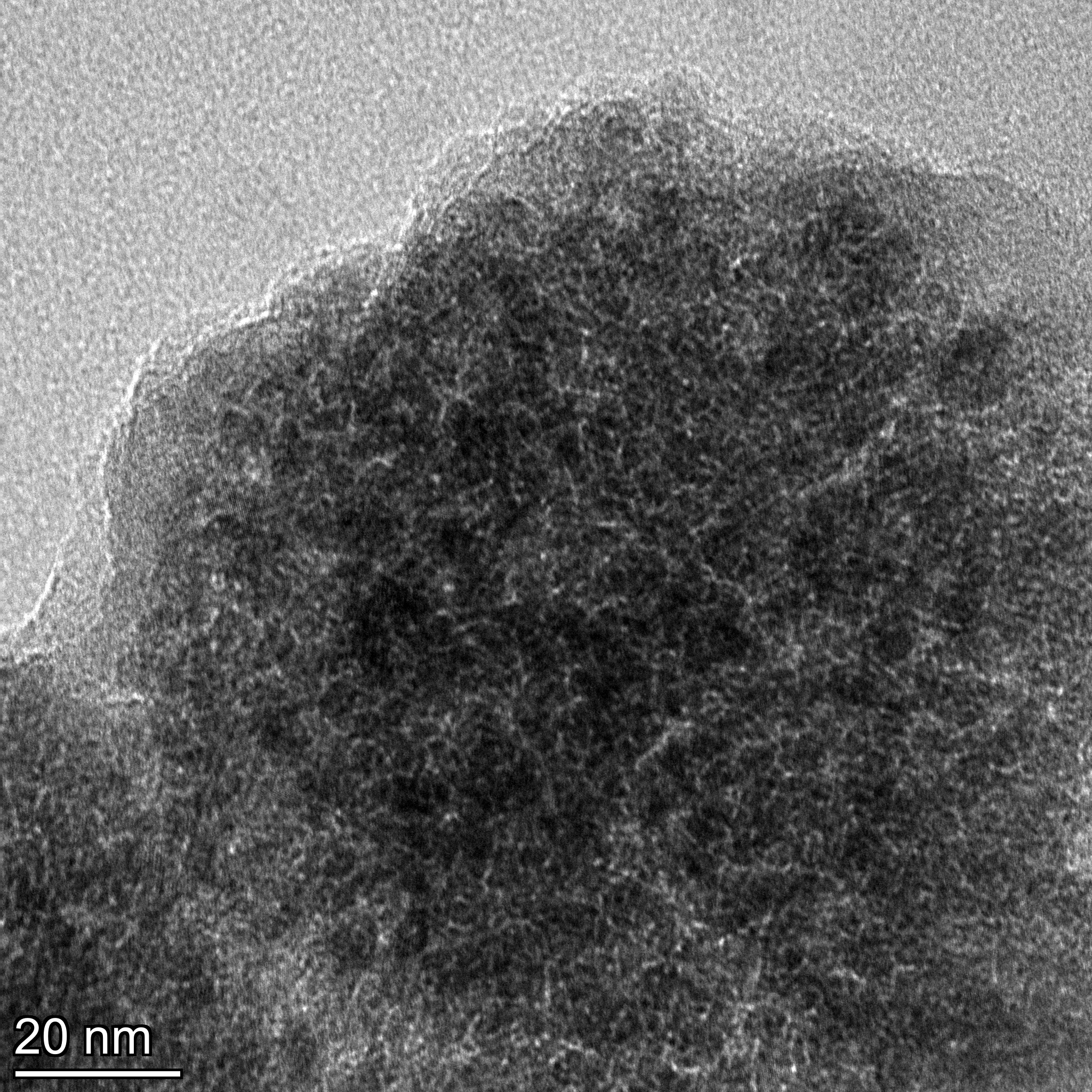

Supplement: Supplemental Information 20 [file peerj-13-19082-s020.jpg]

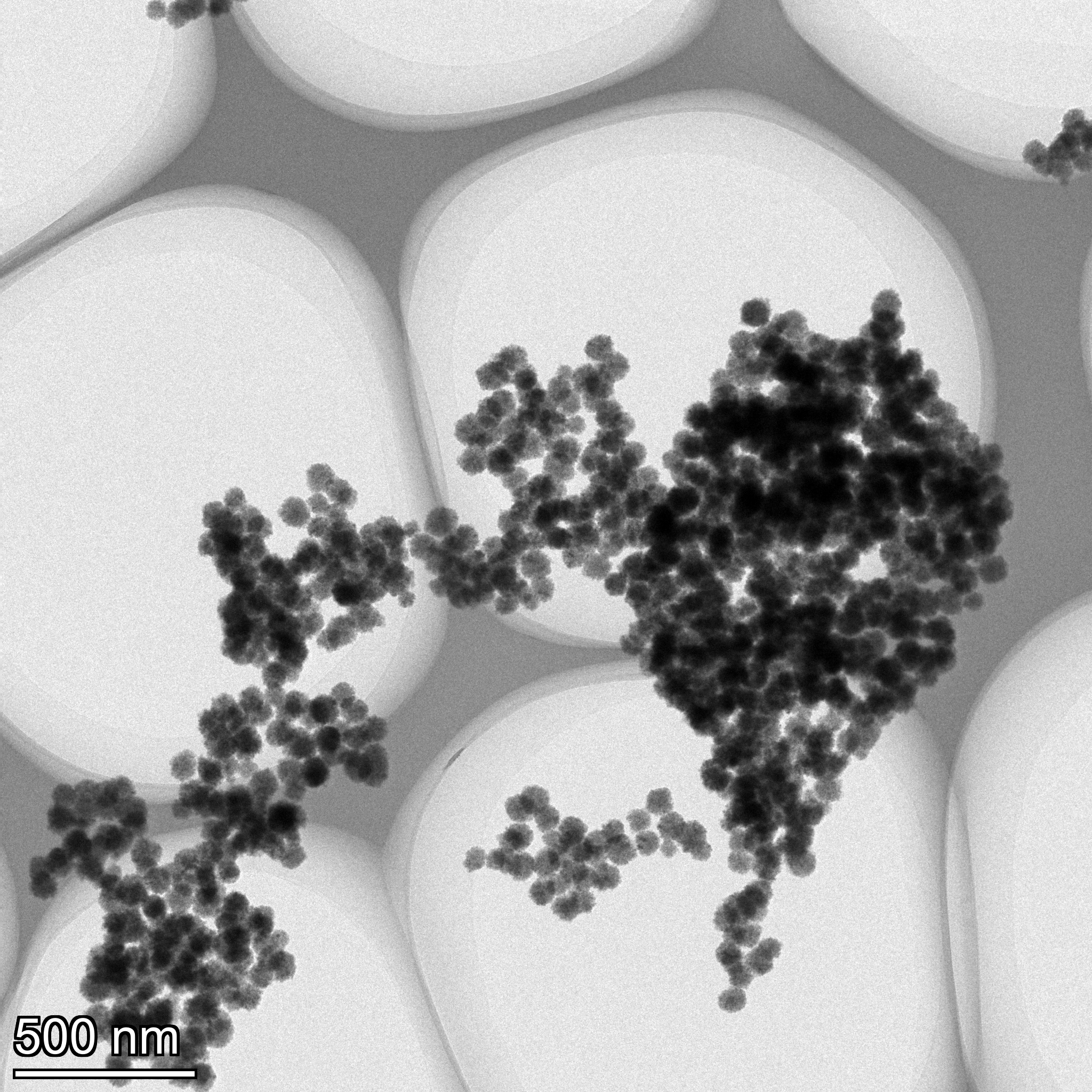

Supplement: Supplemental Information 21 [file peerj-13-19082-s021.jpg]

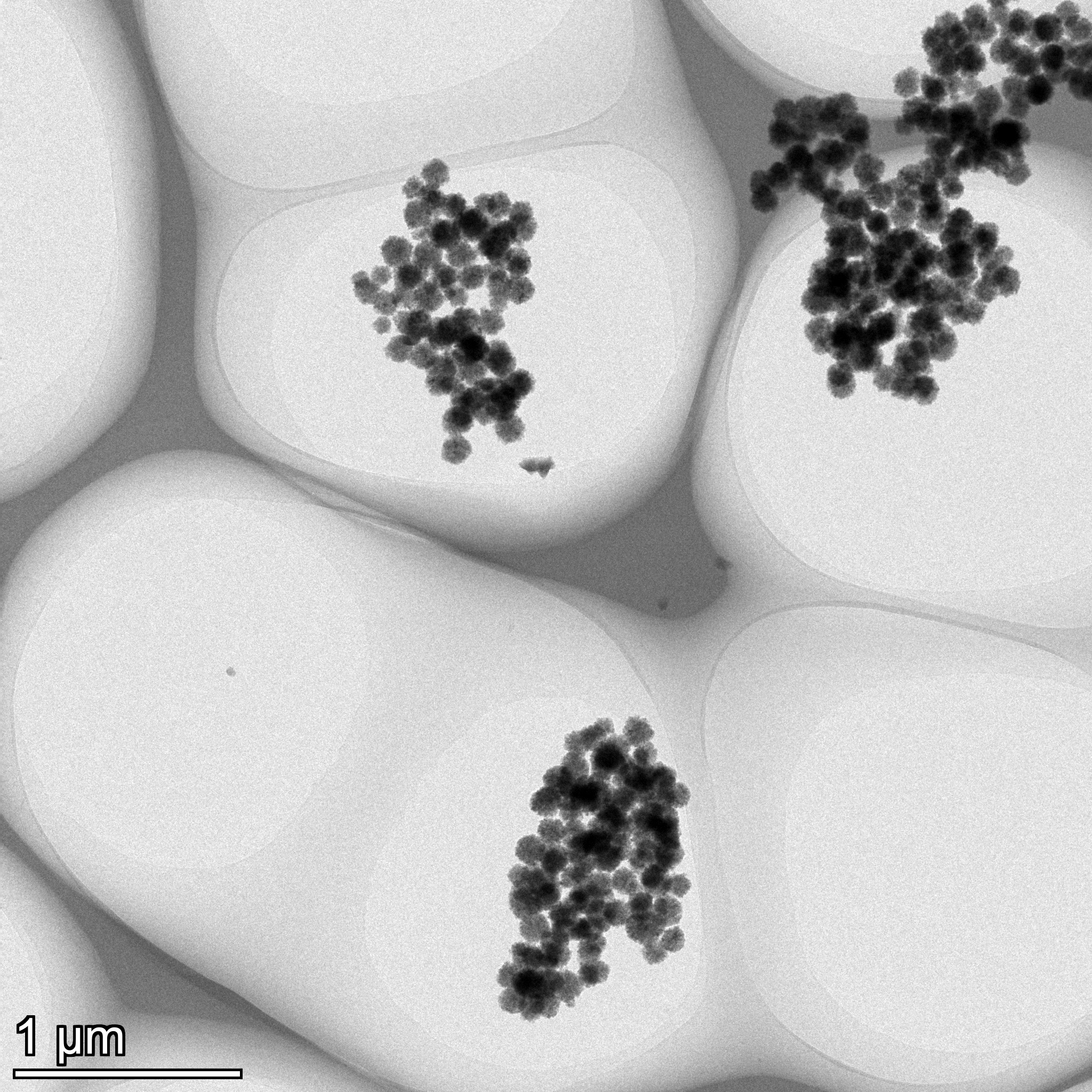

Supplement: Supplemental Information 22 [file peerj-13-19082-s022.jpg]

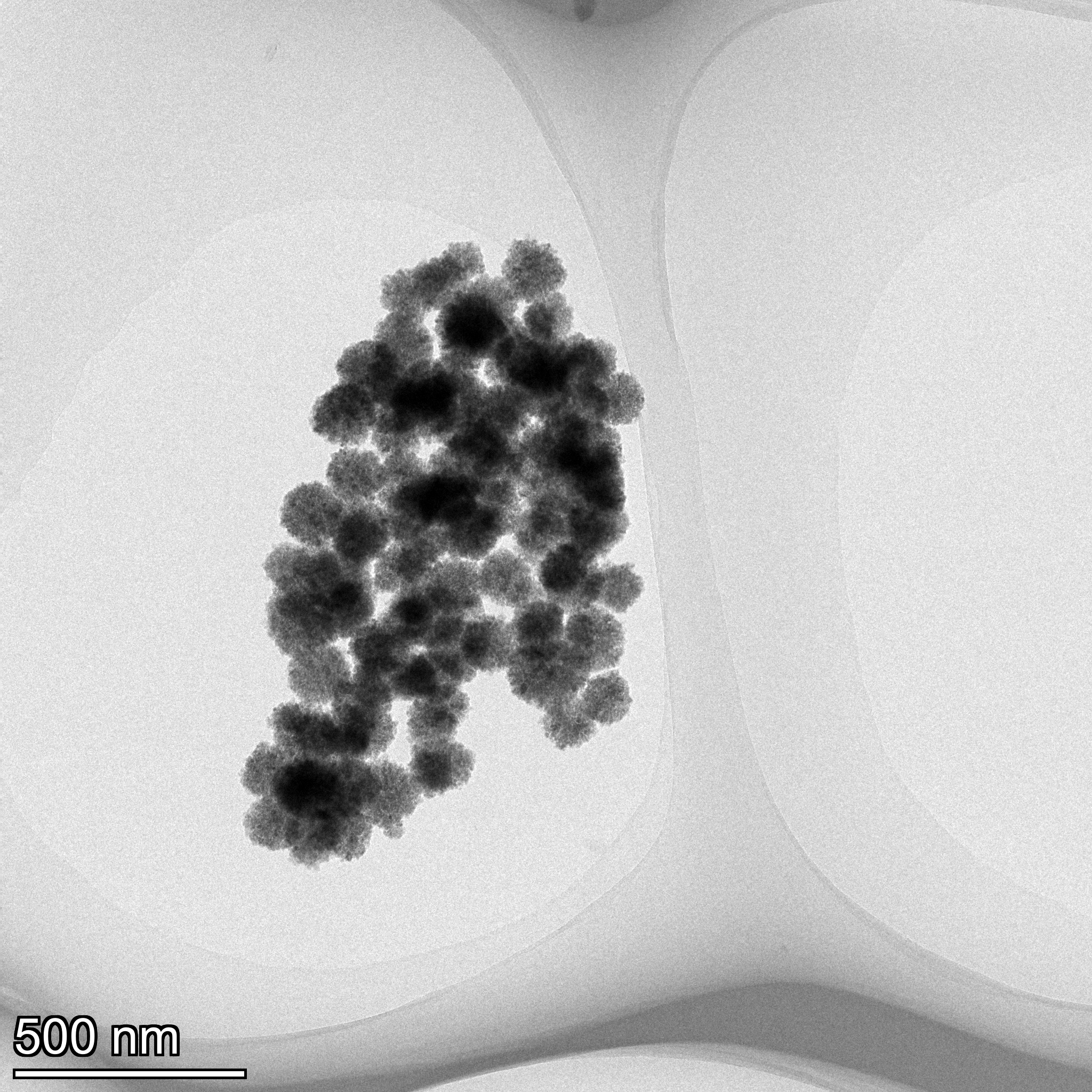

Supplement: Supplemental Information 23 [file peerj-13-19082-s023.jpg]

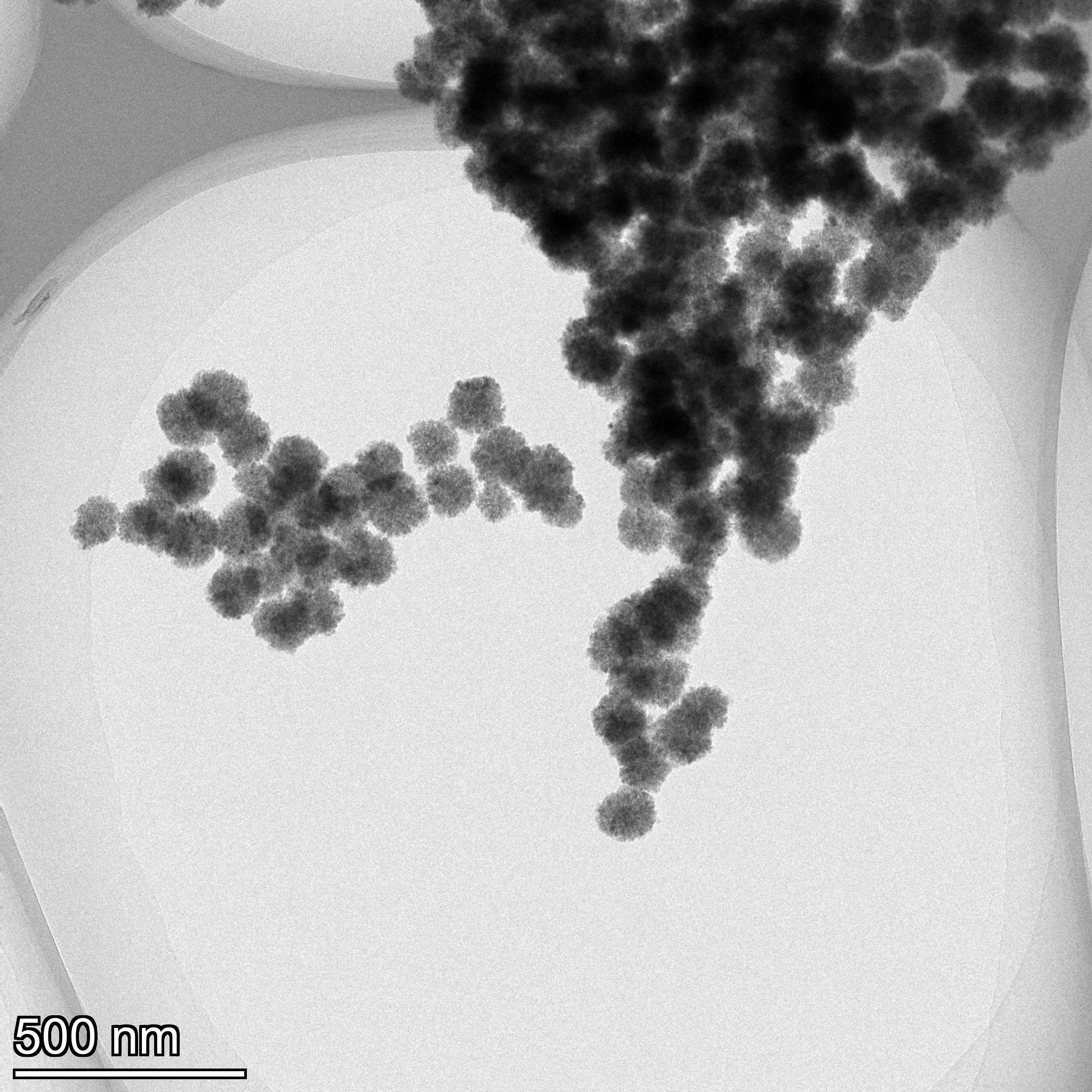

Supplement: Supplemental Information 24 [file peerj-13-19082-s024.jpg]

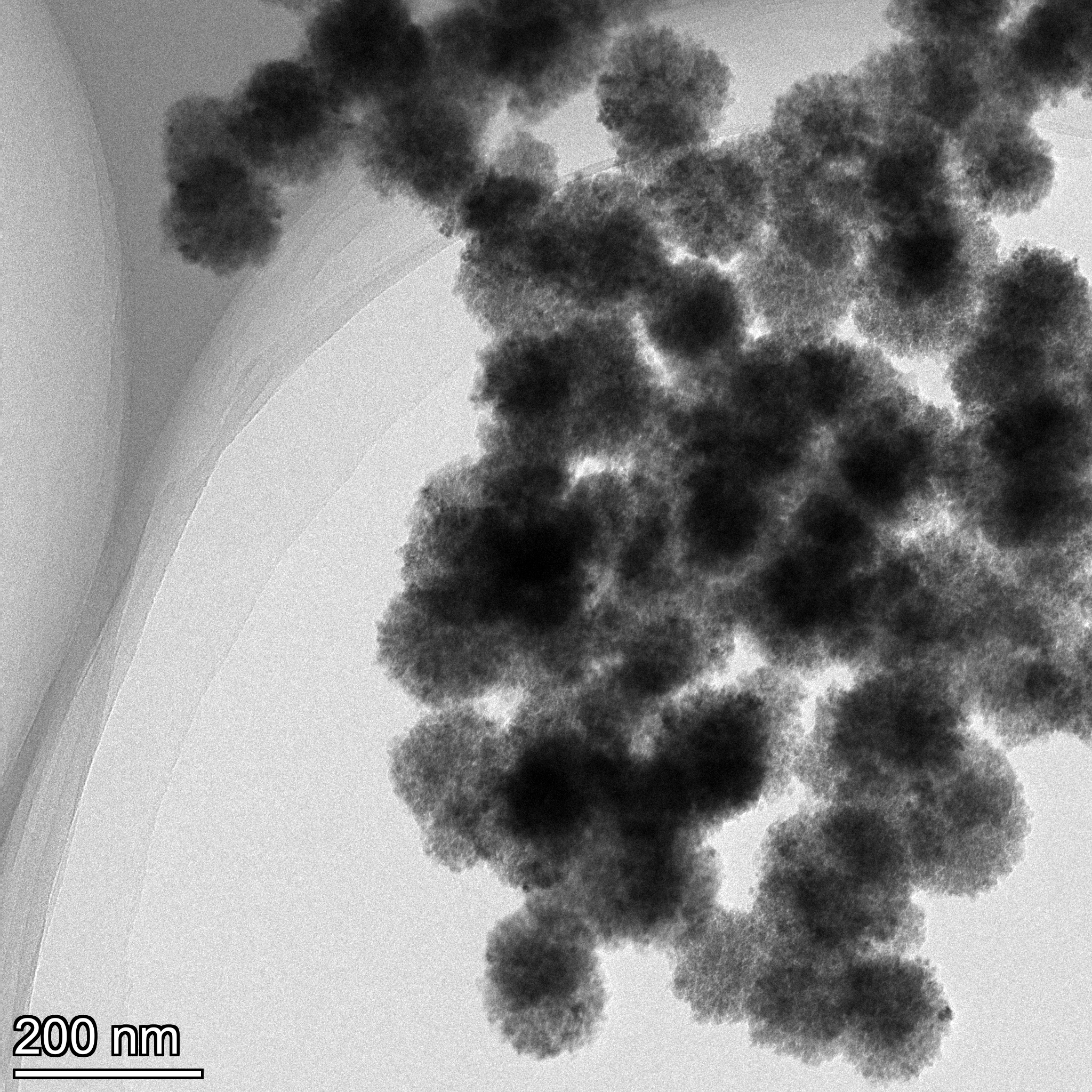

Supplement: Supplemental Information 25 [file peerj-13-19082-s025.jpg]

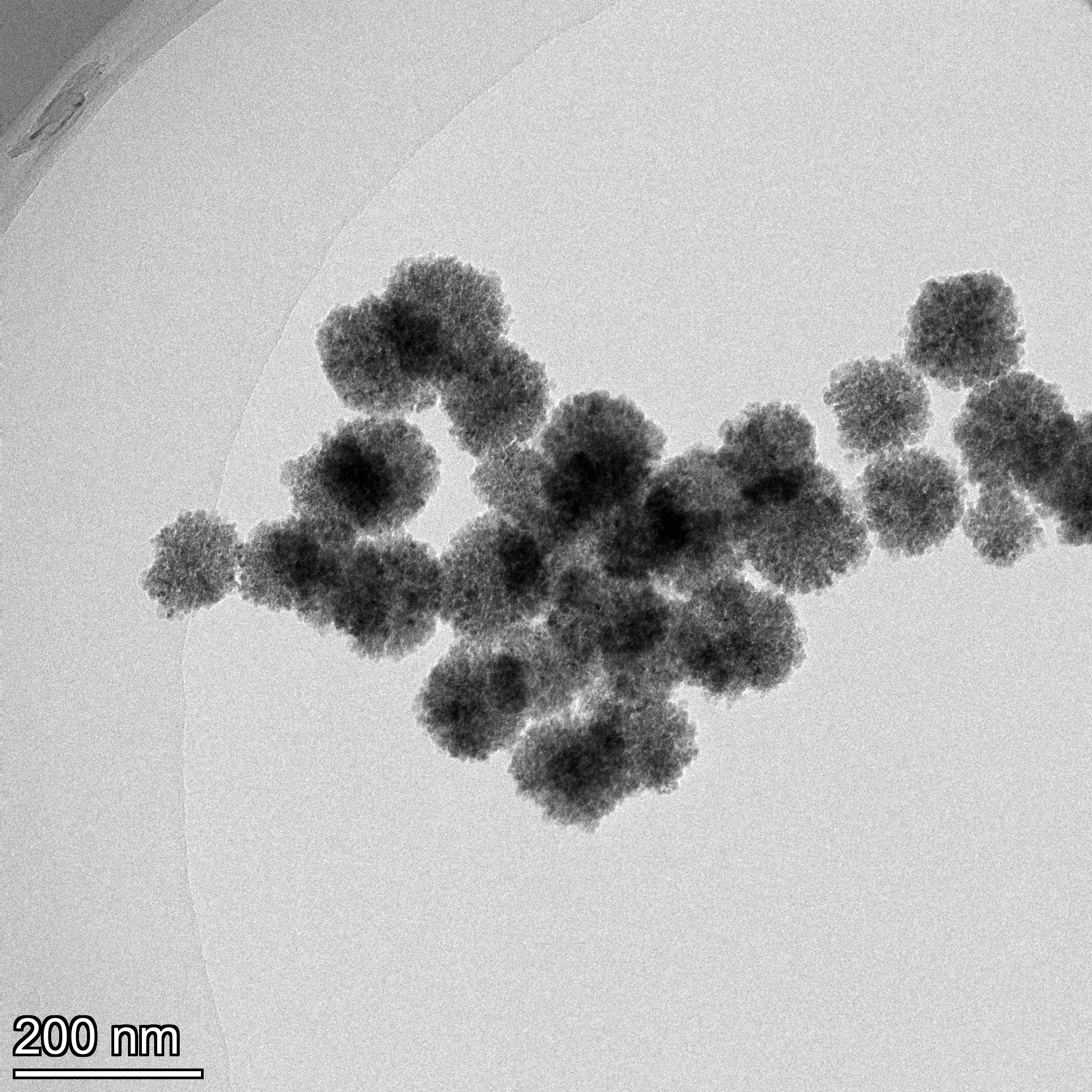

Supplement: Supplemental Information 26 [file peerj-13-19082-s026.jpg]

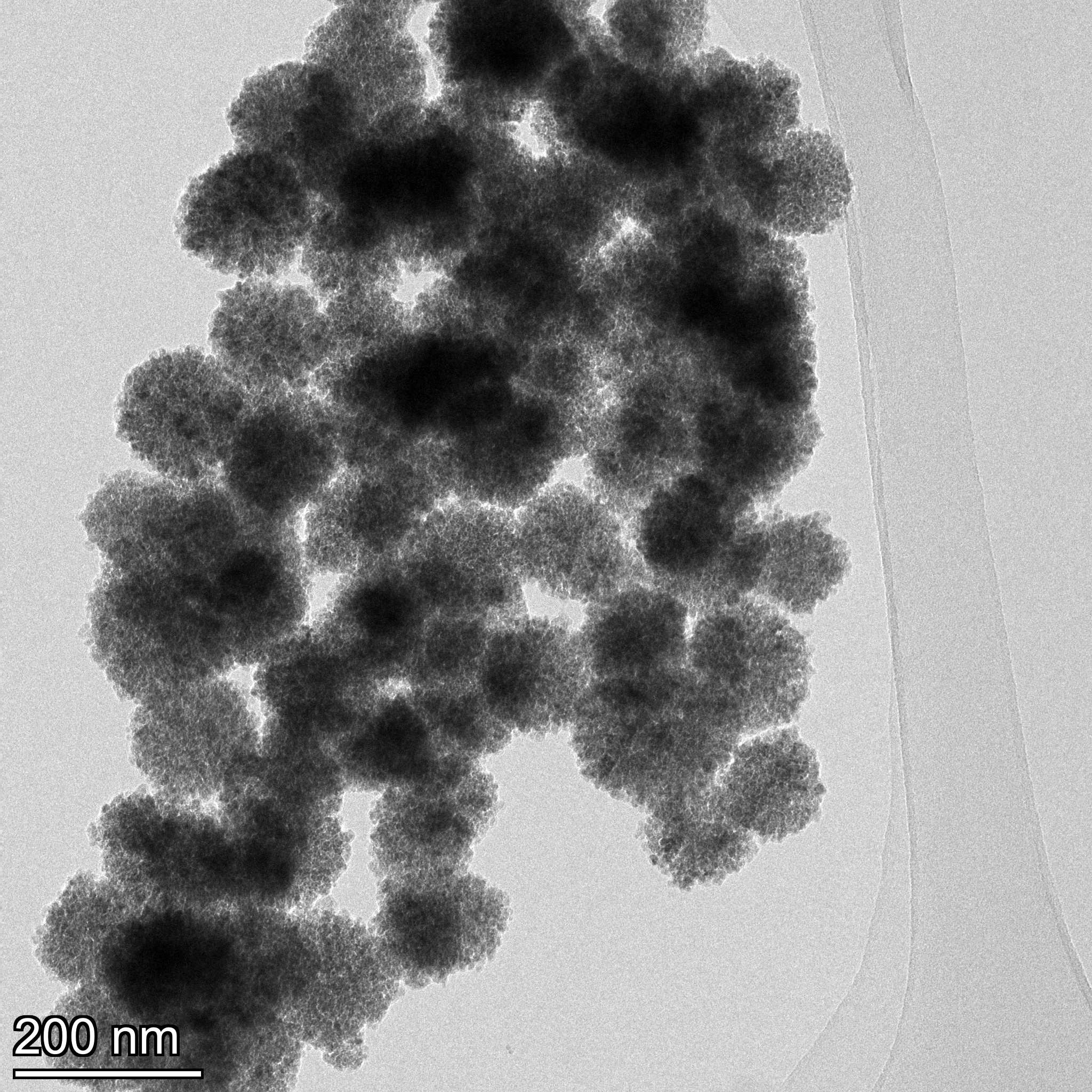

Supplement: Supplemental Information 27 [file peerj-13-19082-s027.jpg]

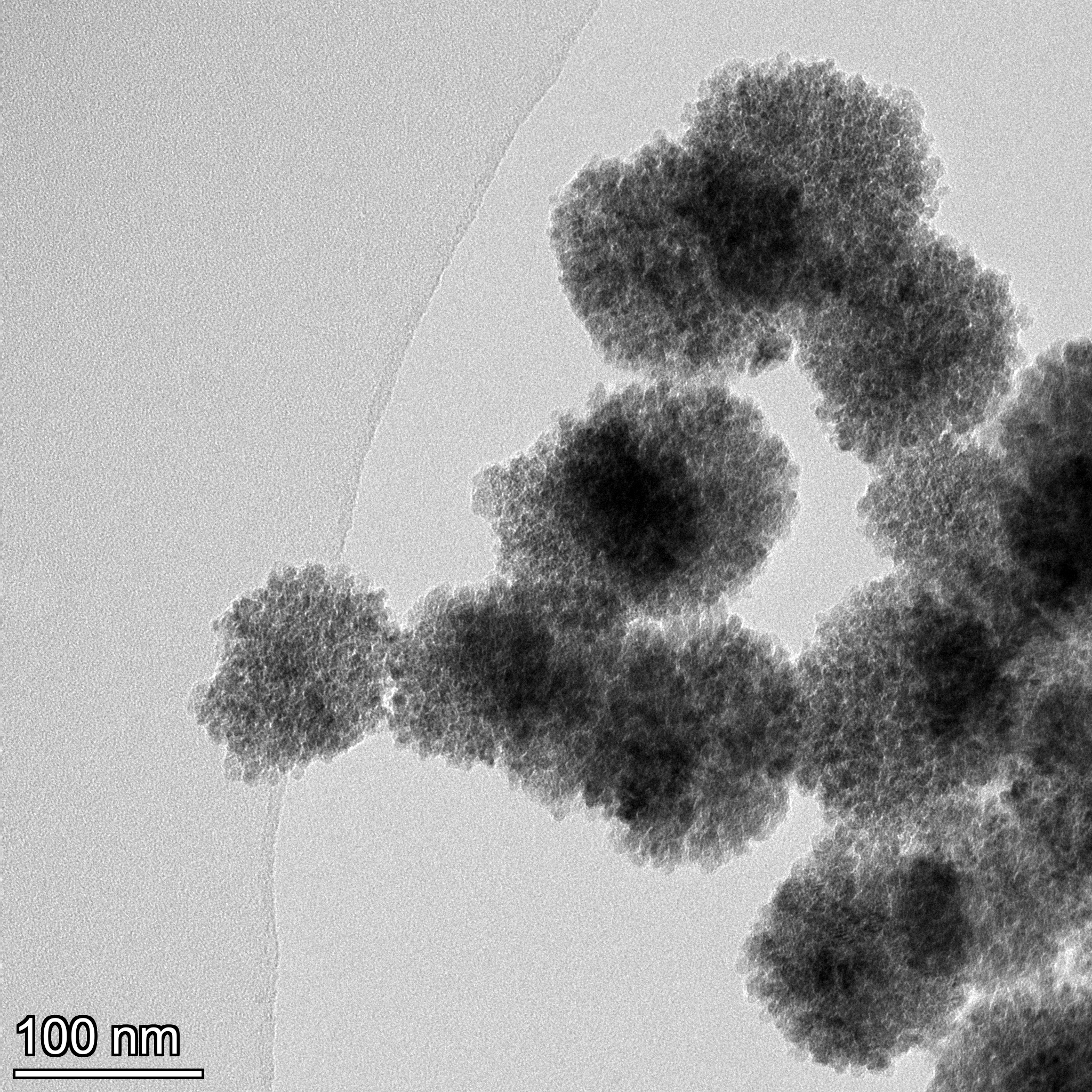

Supplement: Supplemental Information 28 [file peerj-13-19082-s028.jpg]

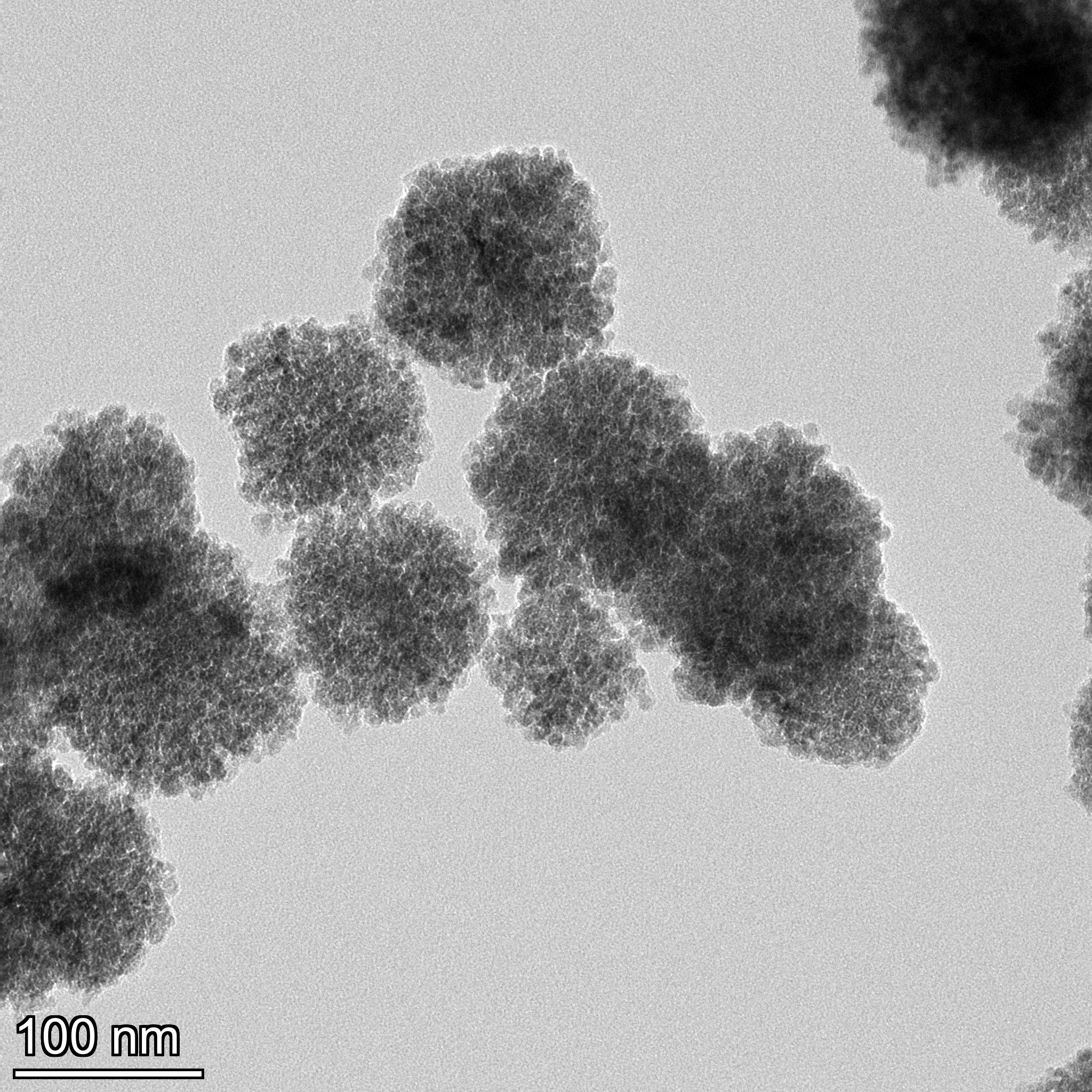

Supplement: Supplemental Information 29 [file peerj-13-19082-s029.jpg]

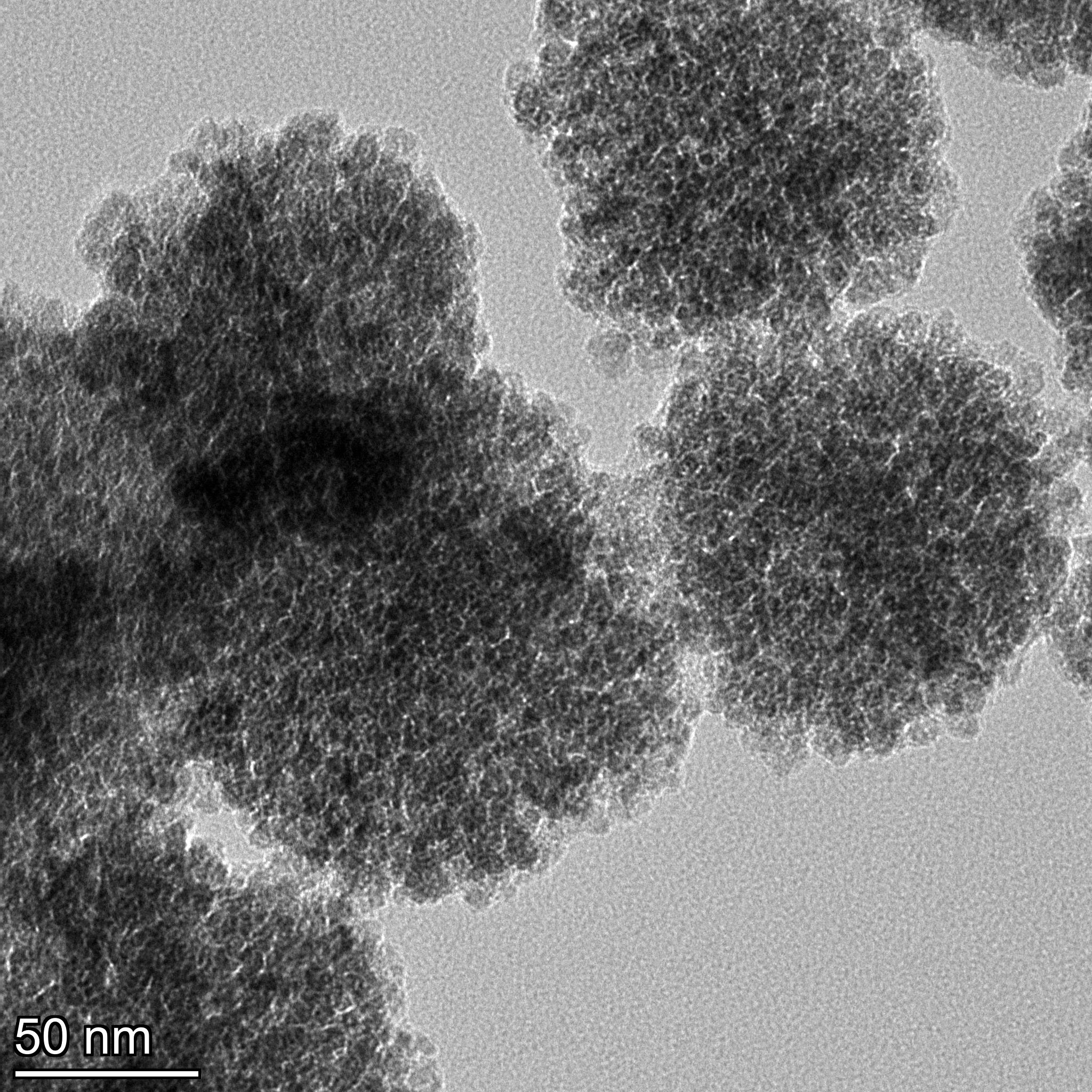

Supplement: Supplemental Information 30 [file peerj-13-19082-s030.jpg]

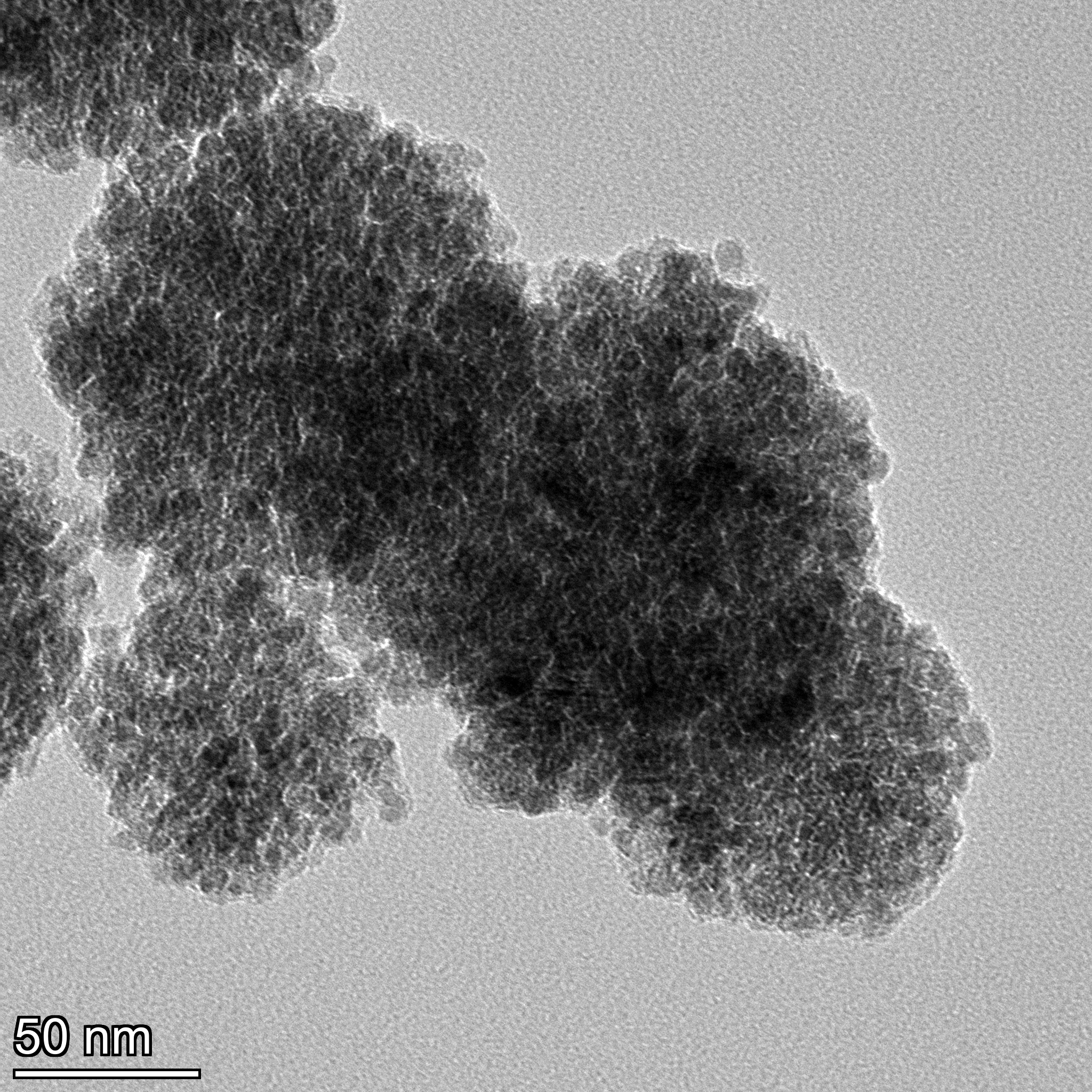

Supplement: Supplemental Information 31 [file peerj-13-19082-s031.jpg]

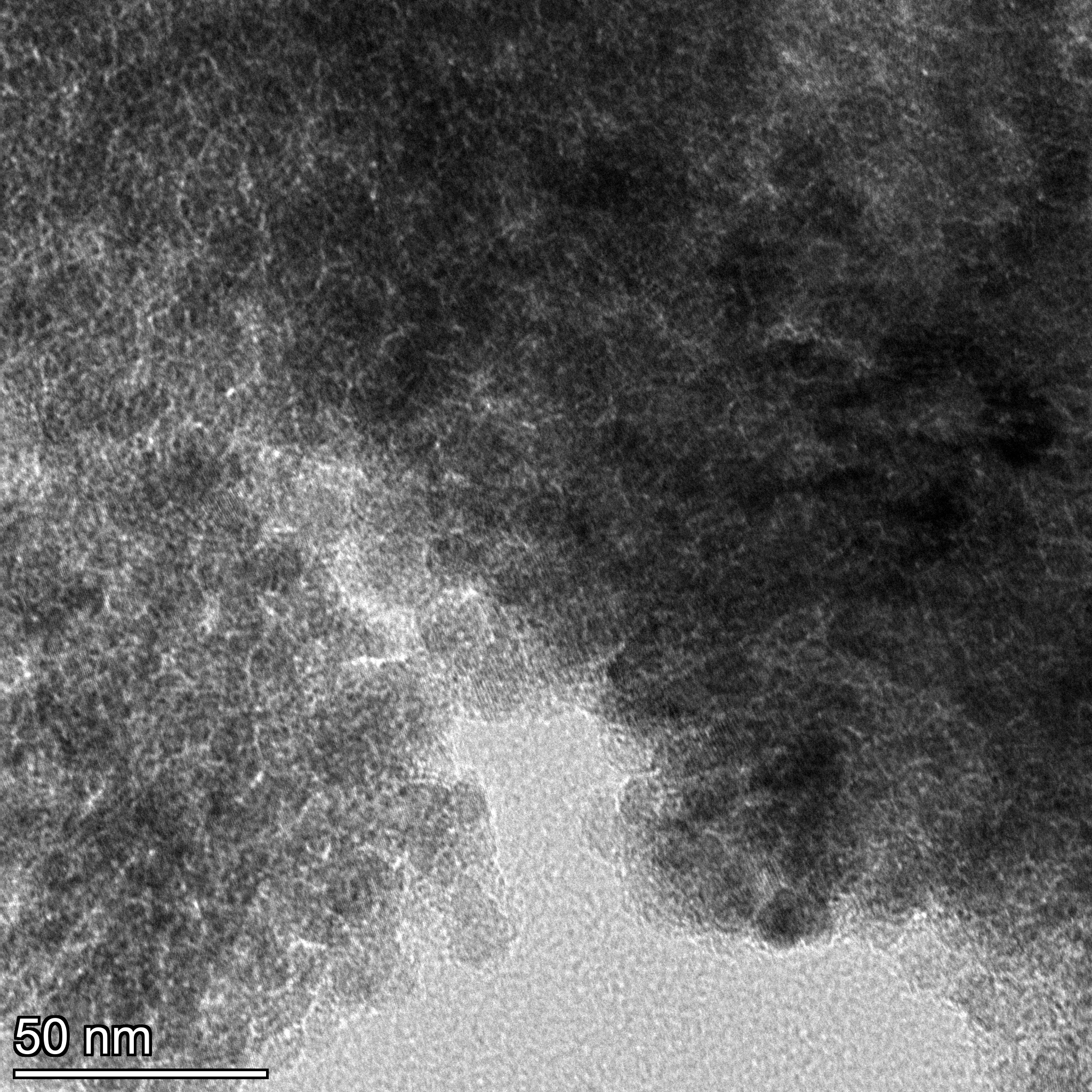

Supplement: Supplemental Information 32 [file peerj-13-19082-s032.jpg]

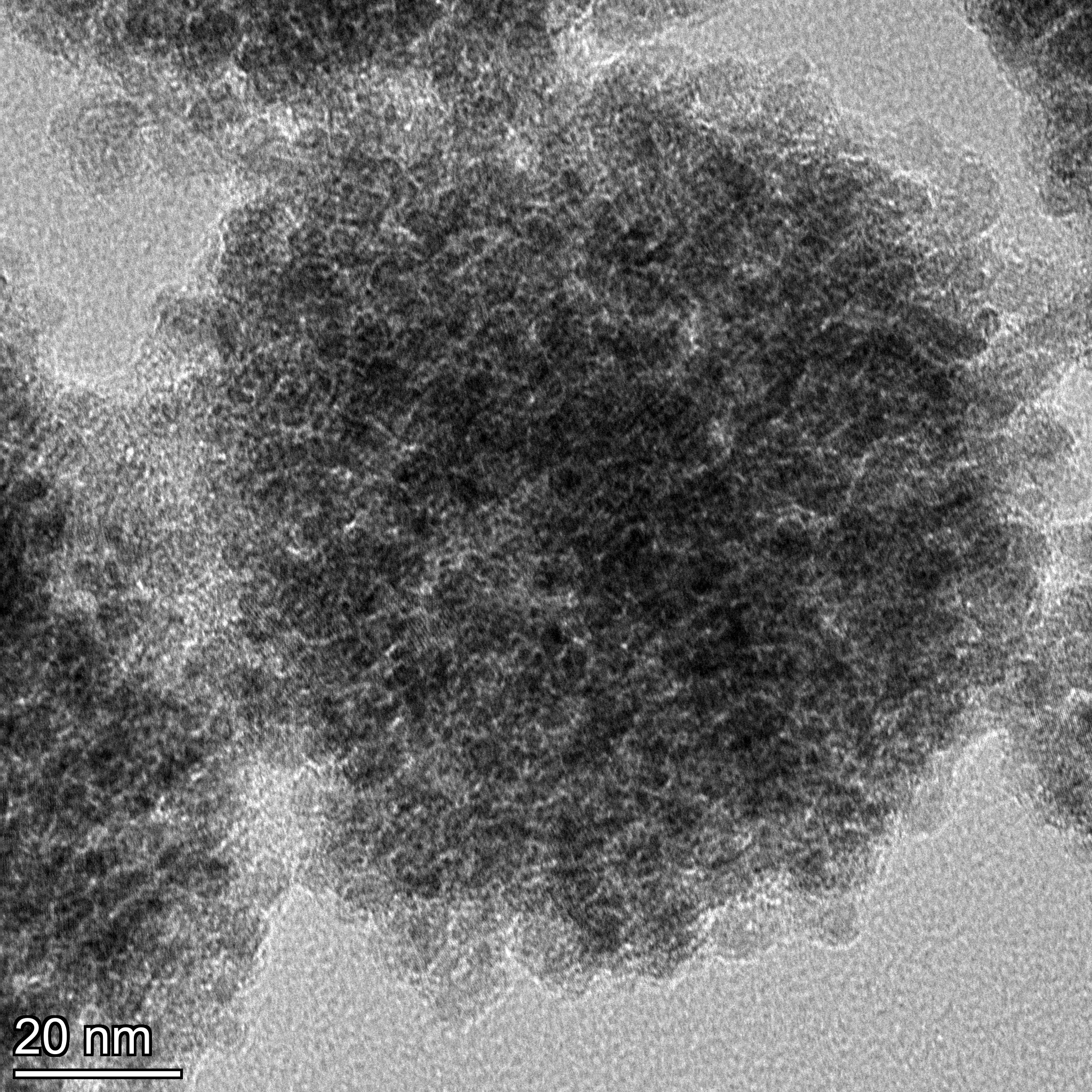

Supplement: Supplemental Information 33 [file peerj-13-19082-s033.jpg]

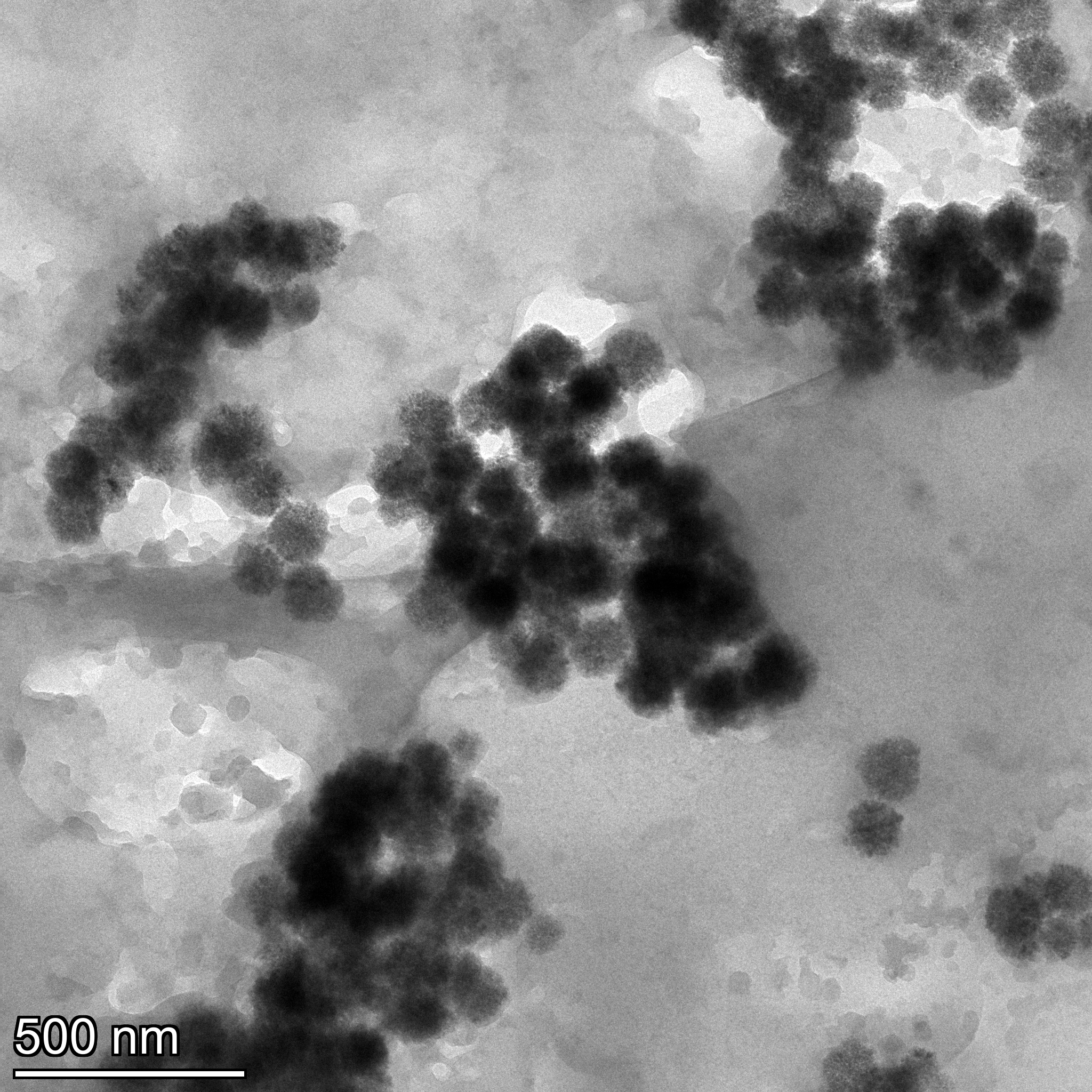

Supplement: Supplemental Information 34 [file peerj-13-19082-s034.jpg]

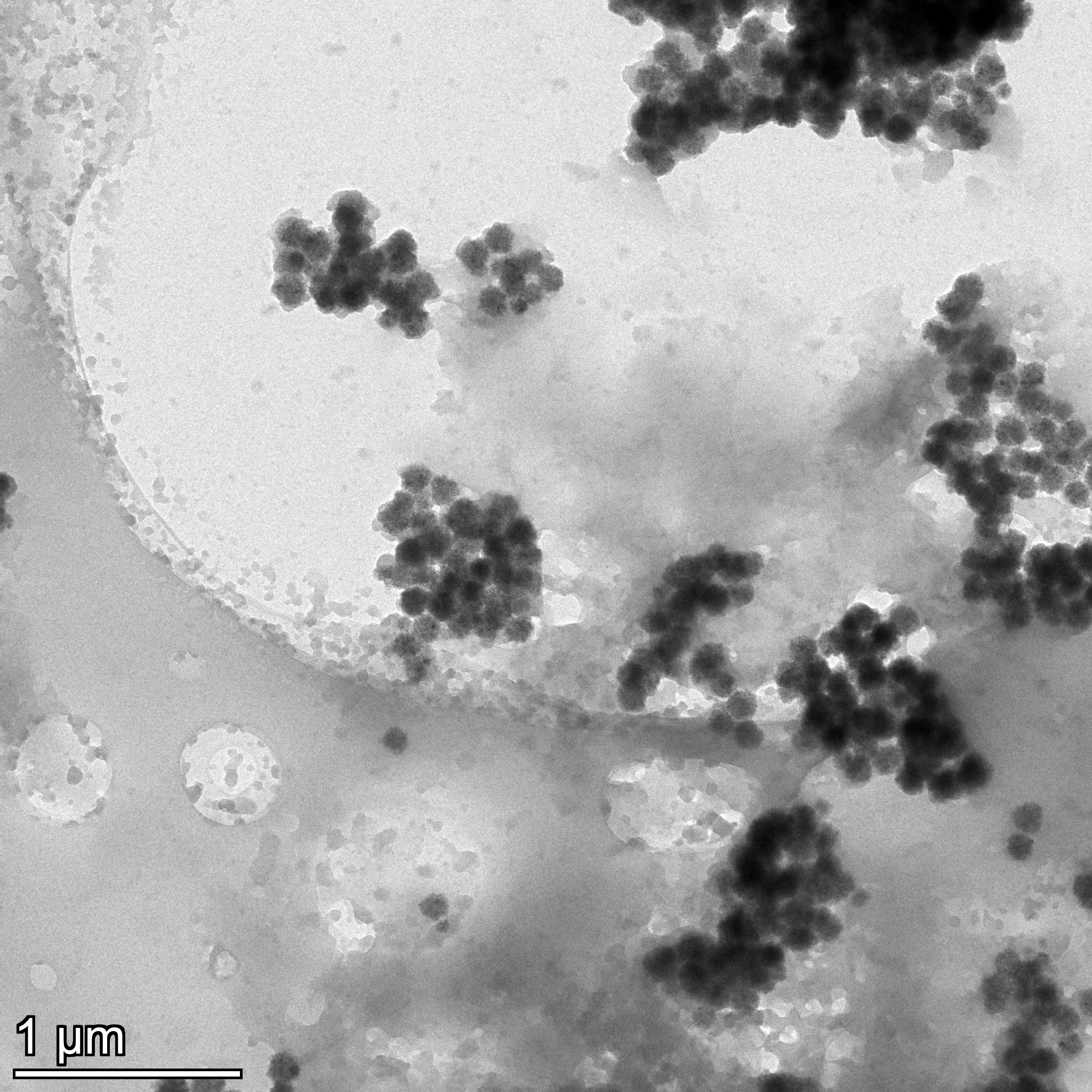

Supplement: Supplemental Information 35 [file peerj-13-19082-s035.jpg]

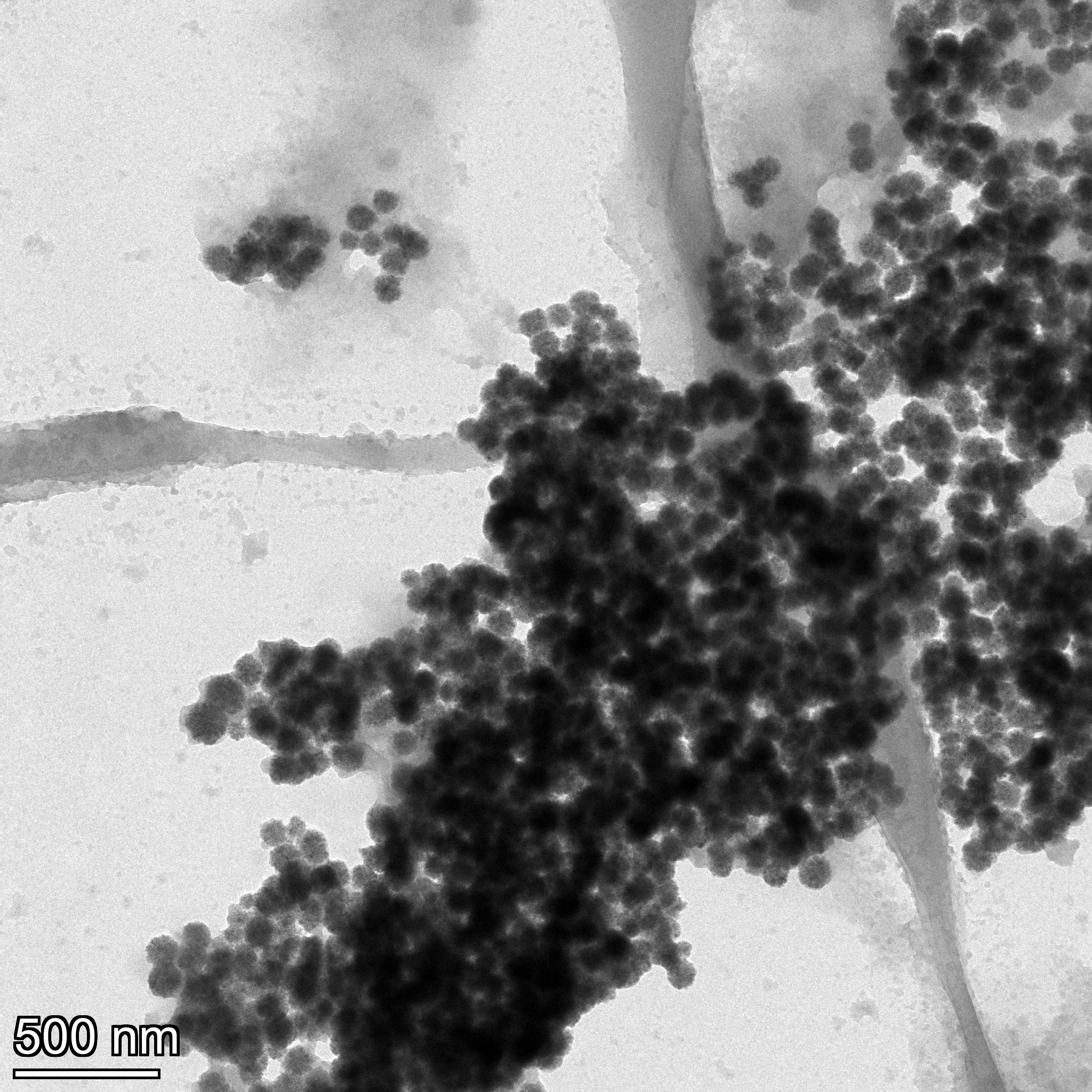

Supplement: Supplemental Information 36 [file peerj-13-19082-s036.jpg]

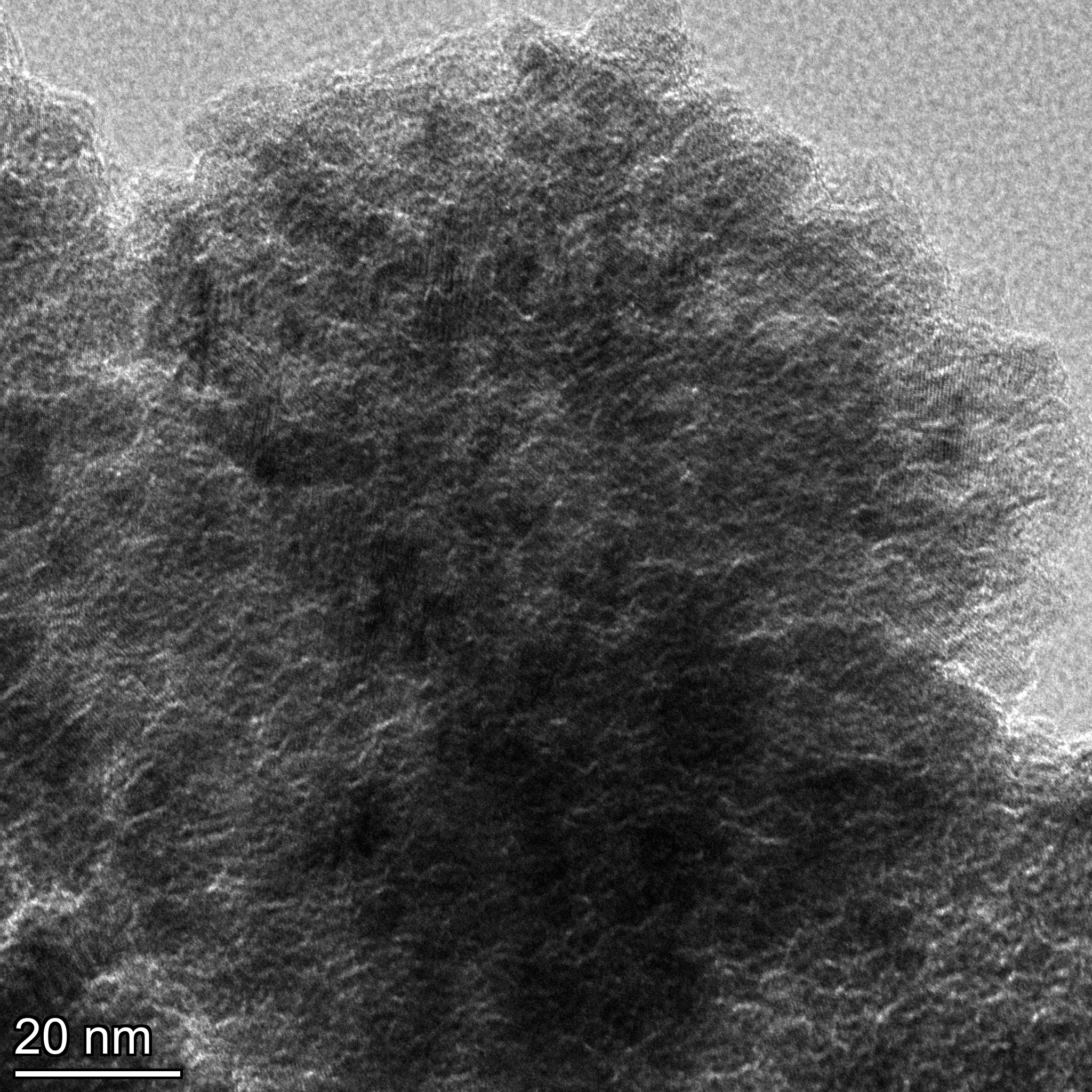

Supplement: Supplemental Information 37 [file peerj-13-19082-s037.jpg]

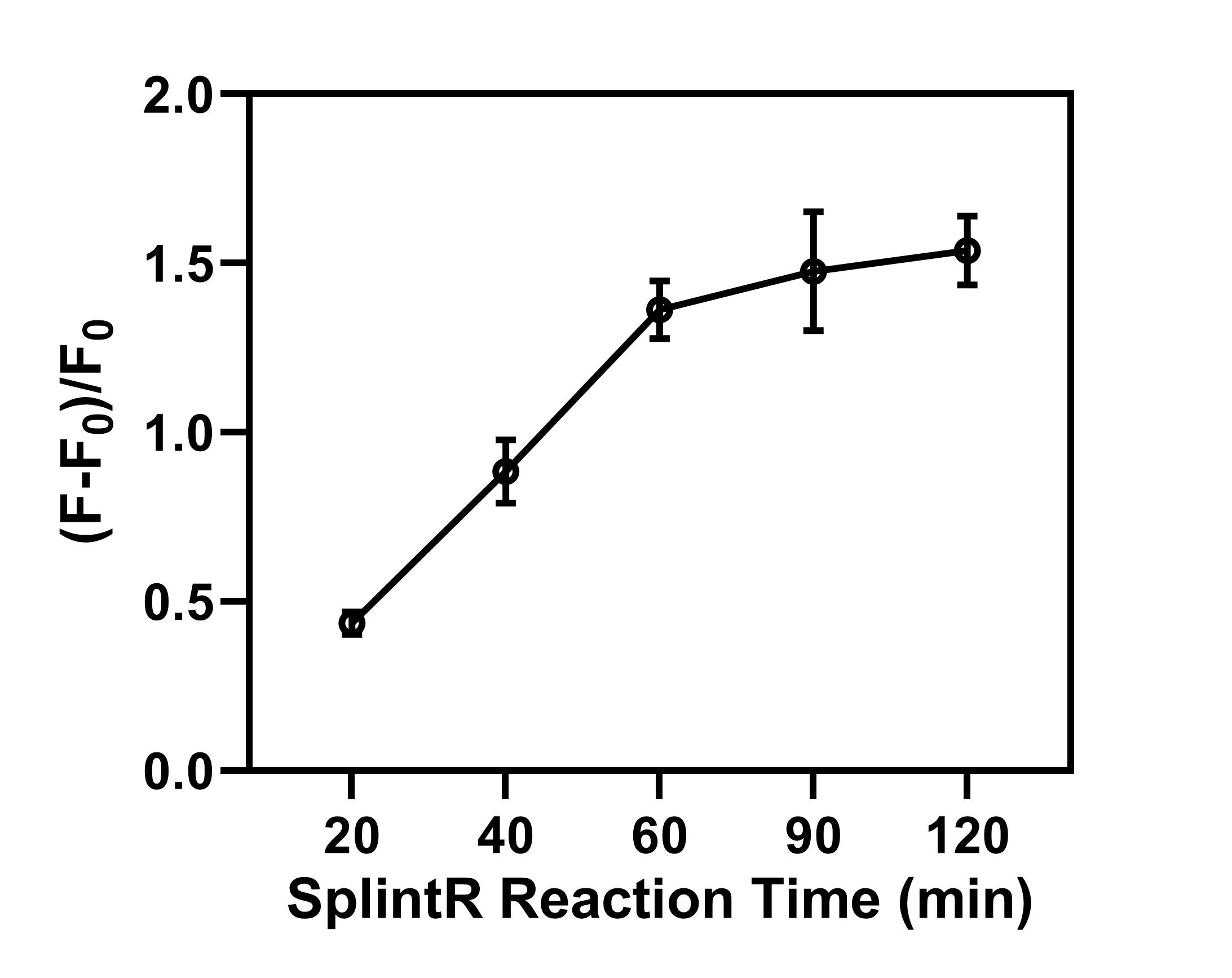

Supplement: Supplemental Information 47 [file peerj-13-19082-s047.jpg]

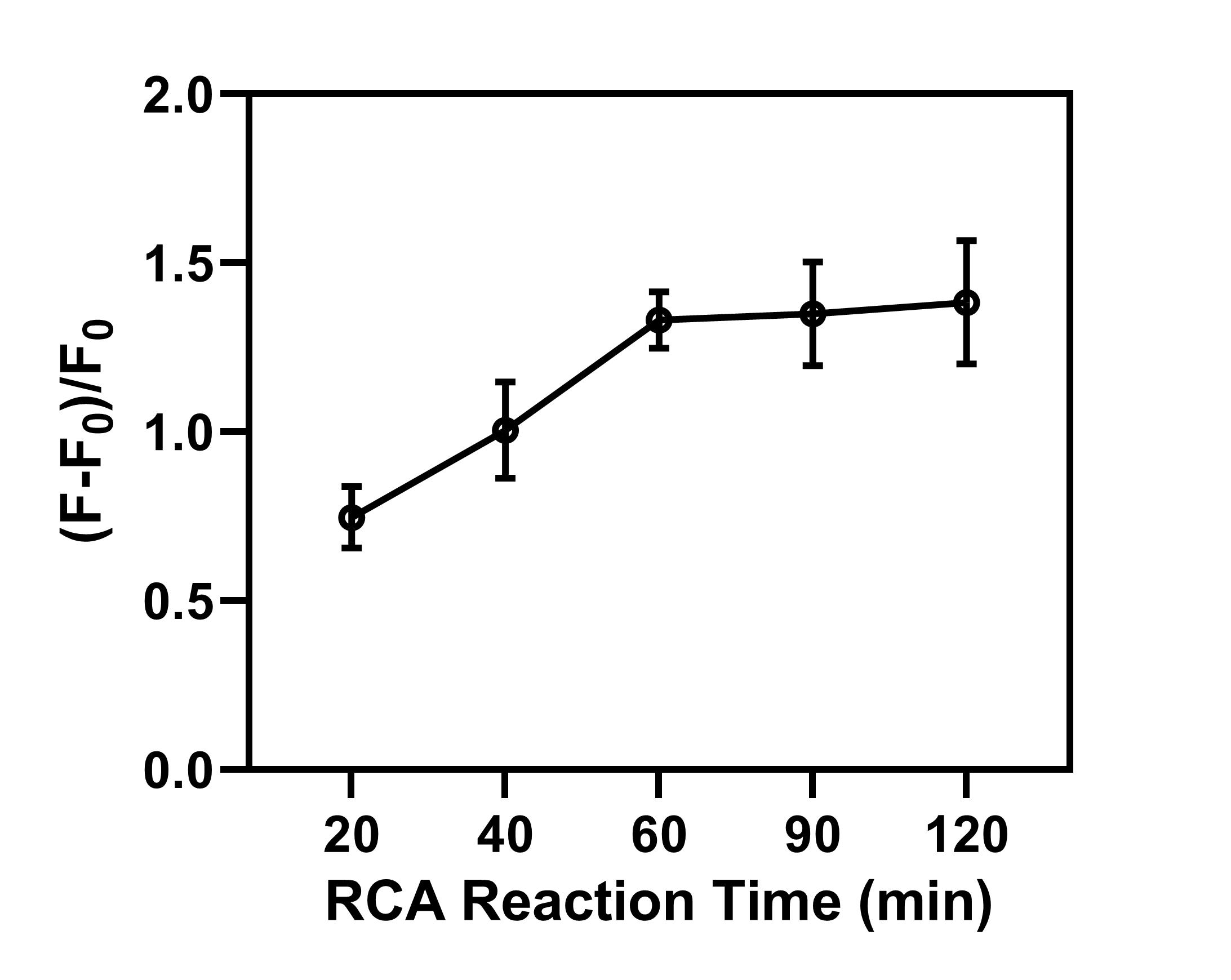

Supplement: Supplemental Information 48 [file peerj-13-19082-s048.jpg]

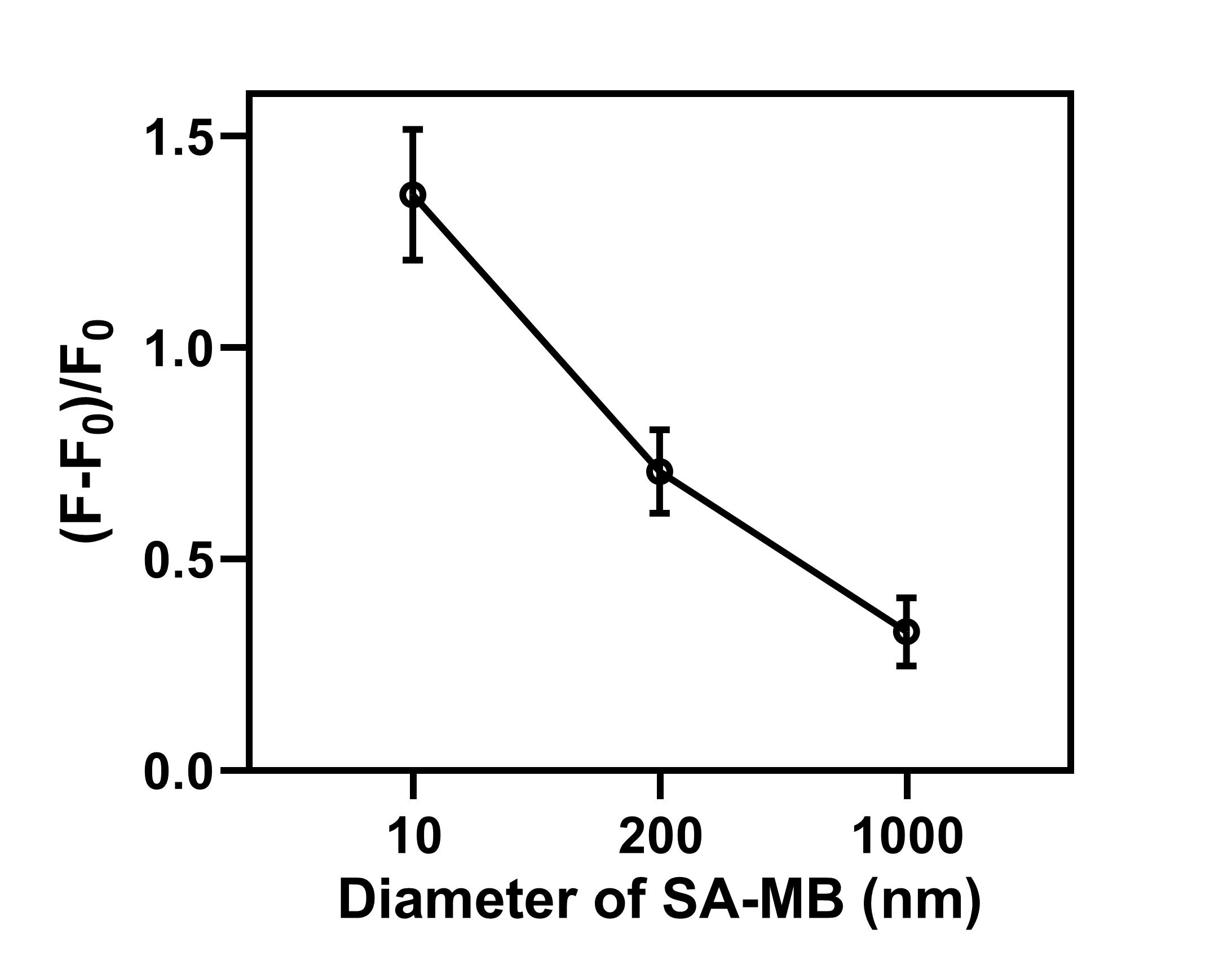

Supplement: Supplemental Information 51 [file peerj-13-19082-s051.jpg]

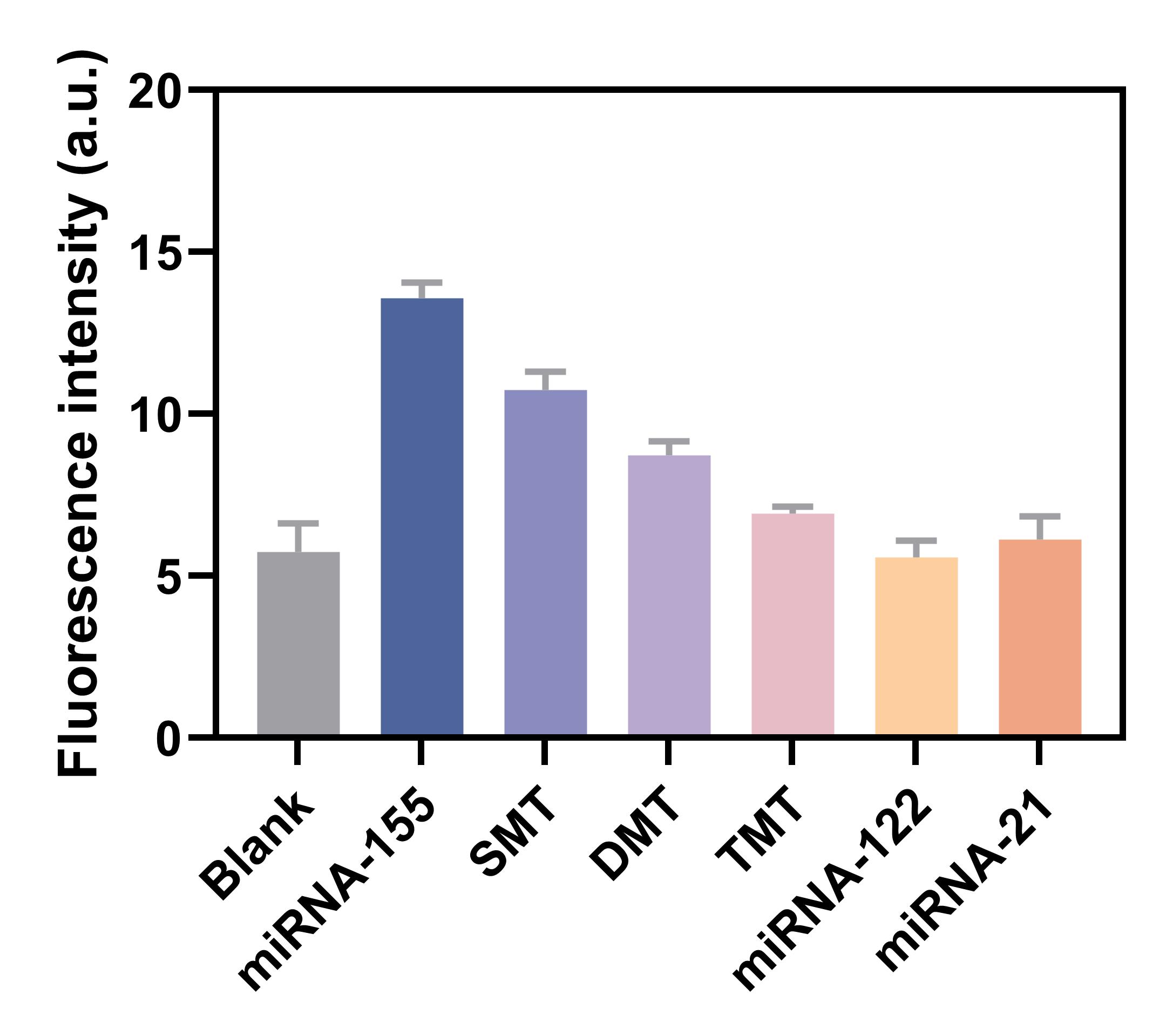

Supplement: Supplemental Information 52 [file peerj-13-19082-s052.jpg]

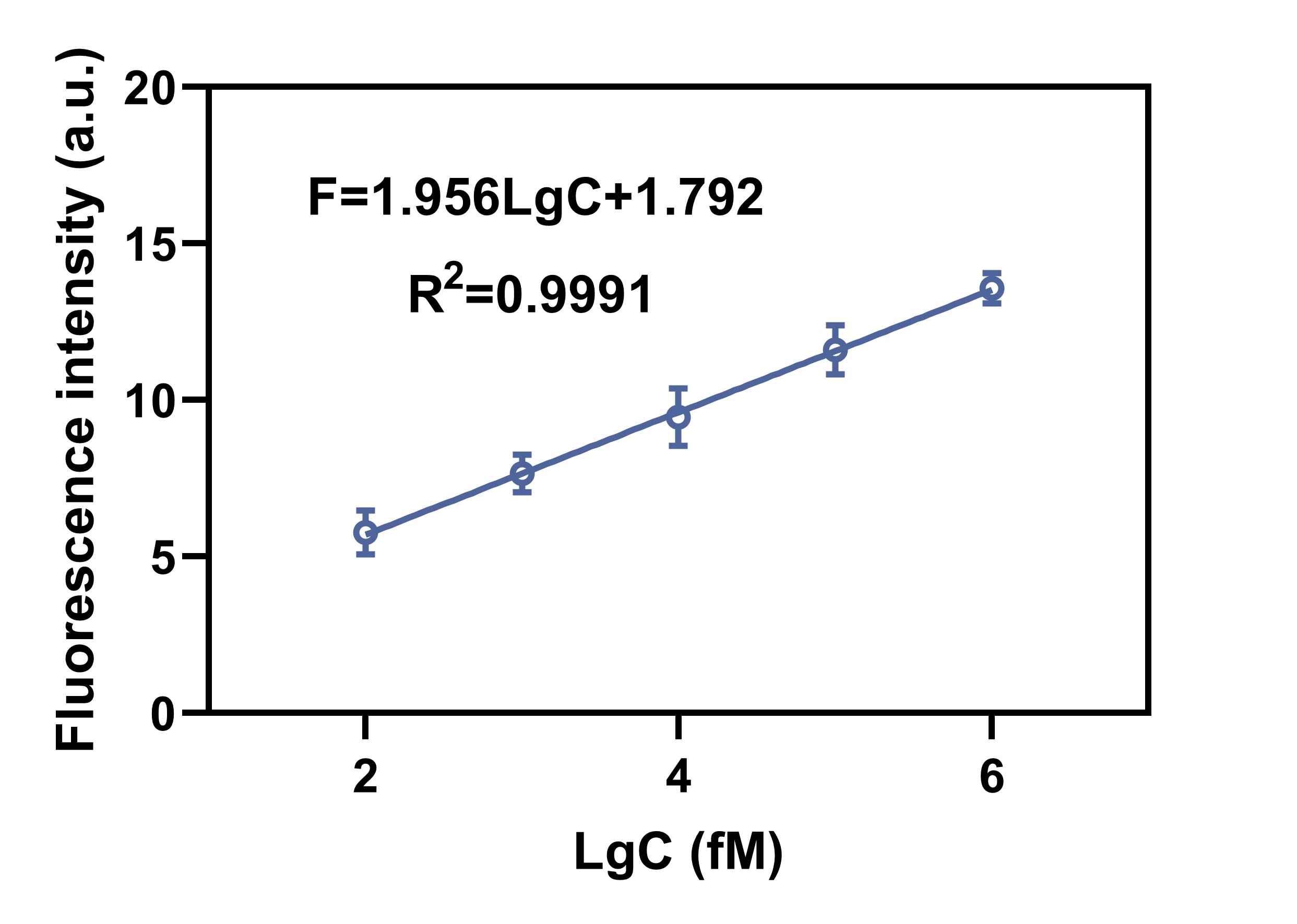

Supplement: Supplemental Information 53 — linearity [file peerj-13-19082-s053.jpg]

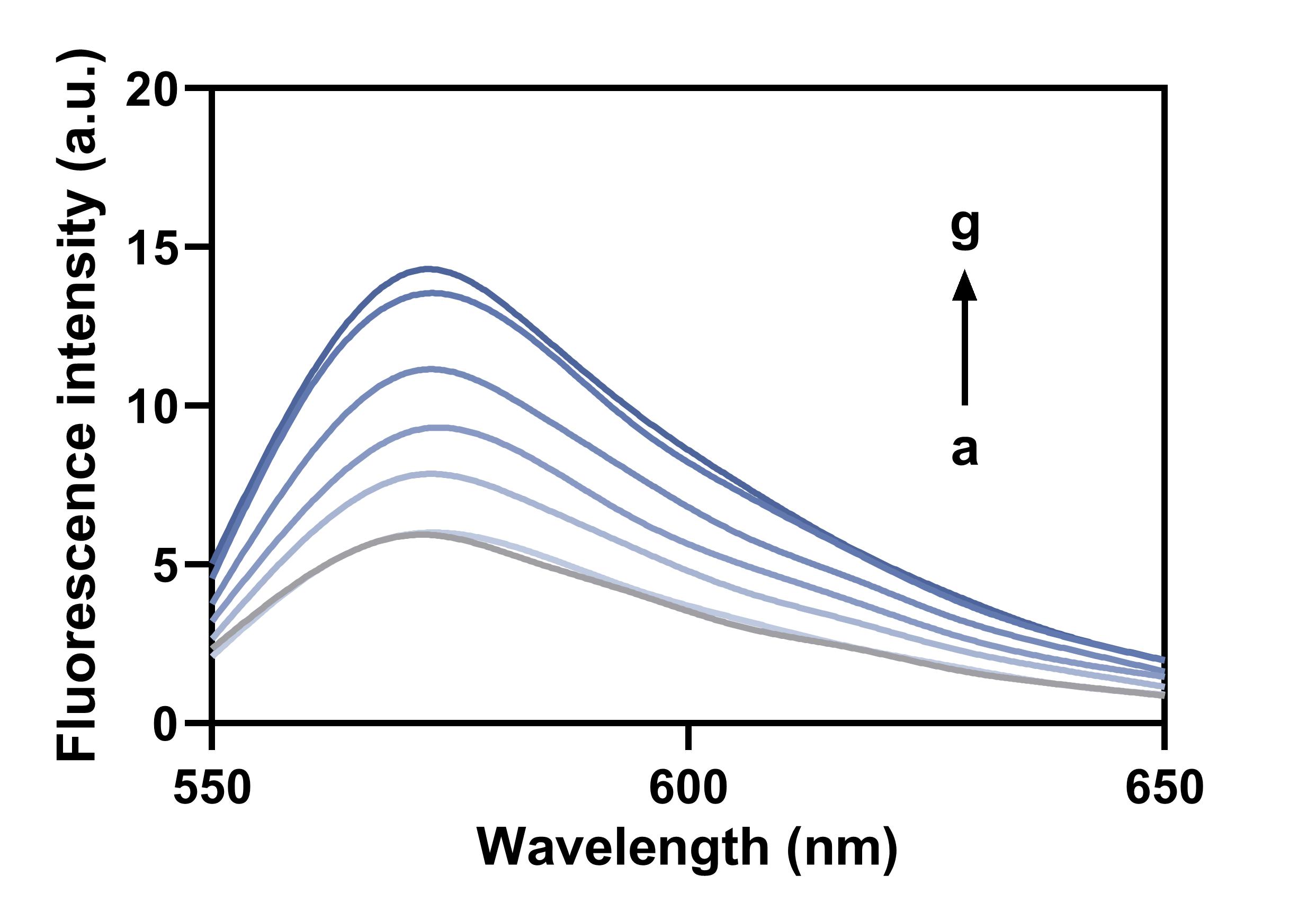

Supplement: Supplemental Information 54 [file peerj-13-19082-s054.jpg]

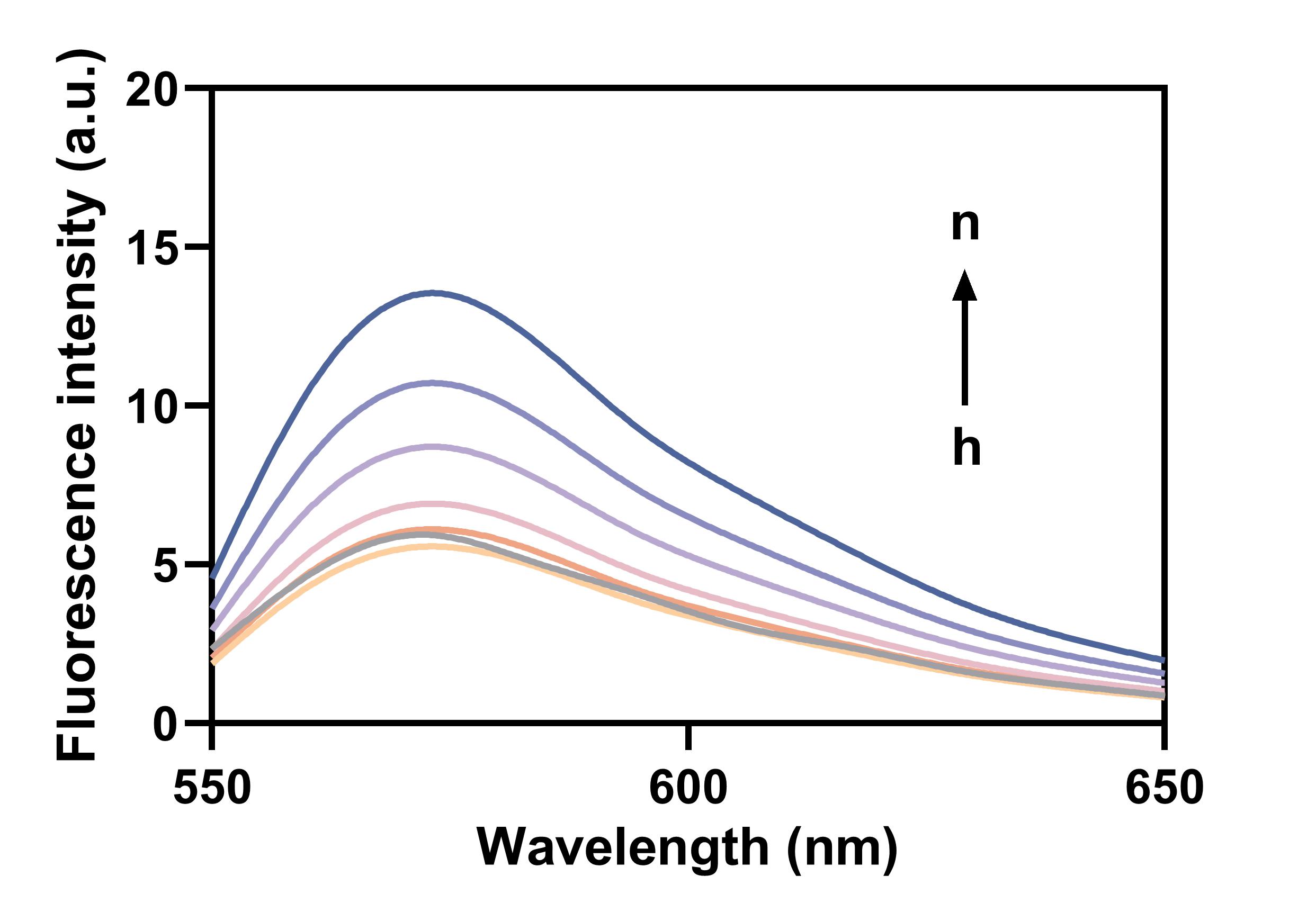

Supplement: Supplemental Information 55 [file peerj-13-19082-s055.jpg]

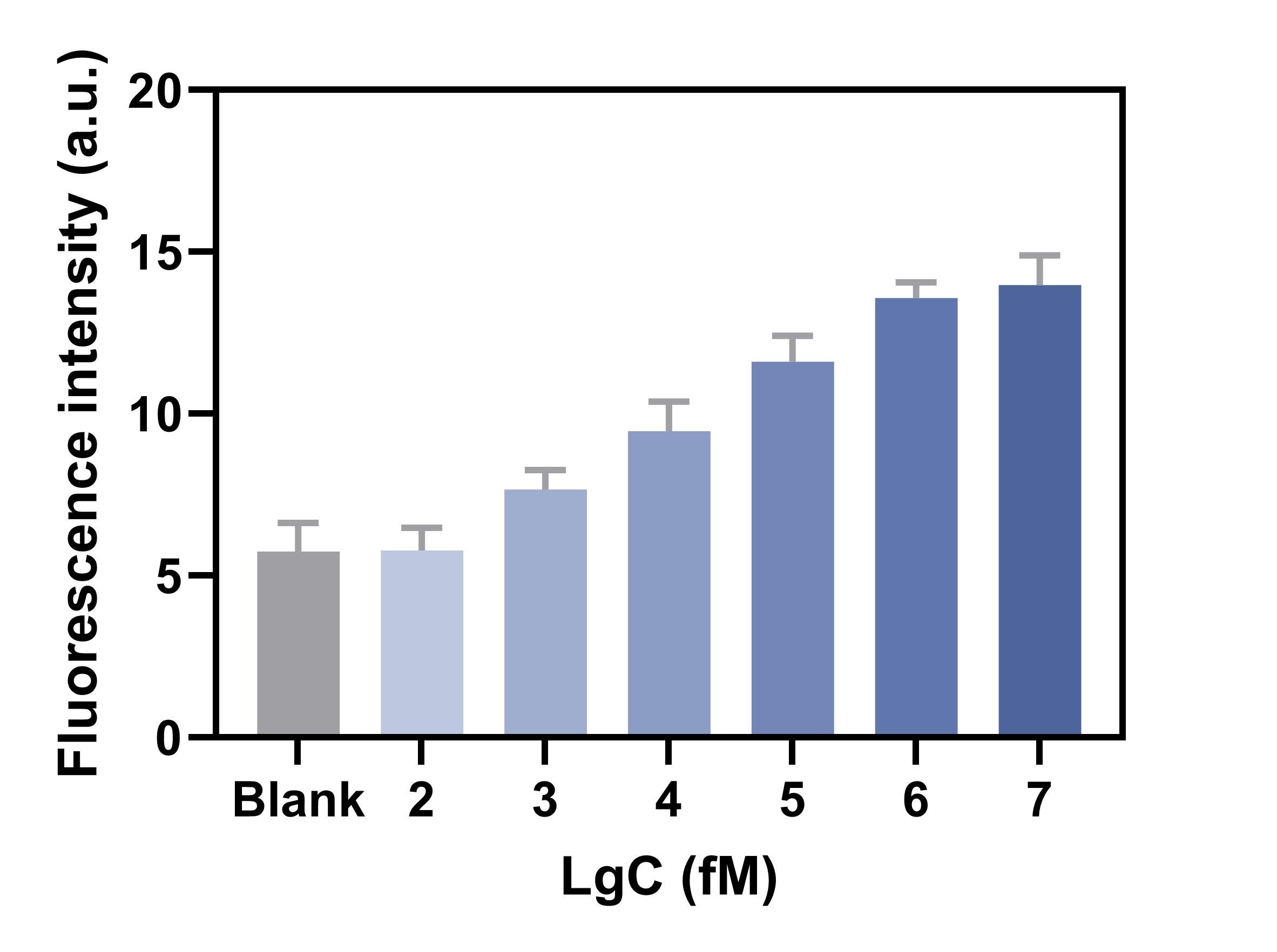

Supplement: Supplemental Information 56 [file peerj-13-19082-s056.jpg]
